# Bone tissue engineering for treating osteonecrosis of the femoral head

**DOI:** 10.1002/EXP.20210105

**Published:** 2023-02-28

**Authors:** Yixin Bian, Tingting Hu, Zehui Lv, Yiming Xu, Yingjie Wang, Han Wang, Wei Zhu, Bin Feng, Ruizheng Liang, Chaoliang Tan, Xisheng Weng

**Affiliations:** ^1^ Department of Orthopedic Surgery State Key Laboratory of Complex Severe and Rare Diseases Peking Union Medical College Hospital Chinese Academy of Medical Science and Peking Union Medical College Beijing China; ^2^ State Key Laboratory of Chemical Resource Engineering Beijing Advanced Innovation Center for Soft Matter Science and Engineering Beijing University of Chemical Technology Beijing China; ^3^ Department of Chemistry City University of Hong Kong Kowloon Hong Kong SAR China

**Keywords:** biomaterials, bone tissue engineering, osteonecrosis of the femoral head, regenerative therapy

## Abstract

Osteonecrosis of the femoral head (ONFH) is a devastating and complicated disease with an unclear etiology. Femoral head‐preserving surgeries have been devoted to delaying and hindering the collapse of the femoral head since their introduction in the last century. However, the isolated femoral head‐preserving surgeries cannot prevent the natural progression of ONFH, and the combination of autogenous or allogeneic bone grafting often leads to many undesired complications. To tackle this dilemma, bone tissue engineering has been widely developed to compensate for the deficiencies of these surgeries. During the last decades, great progress has been made in ingenious bone tissue engineering for ONFH treatment. Herein, we comprehensively summarize the state‐of‐the‐art progress made in bone tissue engineering for ONFH treatment. The definition, classification, etiology, diagnosis, and current treatments of ONFH are first described. Then, the recent progress in the development of various bone‐repairing biomaterials, including bioceramics, natural polymers, synthetic polymers, and metals, for treating ONFH is presented. Thereafter, regenerative therapies for ONFH treatment are also discussed. Finally, we give some personal insights on the current challenges of these therapeutic strategies in the clinic and the future development of bone tissue engineering for ONFH treatment.

## Graphical abstract

Great progress has been made in ingenious bone tissue engineering for osteonecrosis of the femoral head (ONFH) treatment in the last decades. In this review, we comprehensively summarize the state‐of‐the‐art progress made in bone tissue engineering for ONFH treatment. We also propose insights on the current challenges of these advances in the clinic and the future development of bone tissue engineering for ONFH treatment.

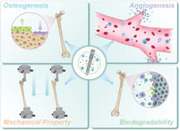

## 1 INTRODUCTION

**FIGURE 1:**
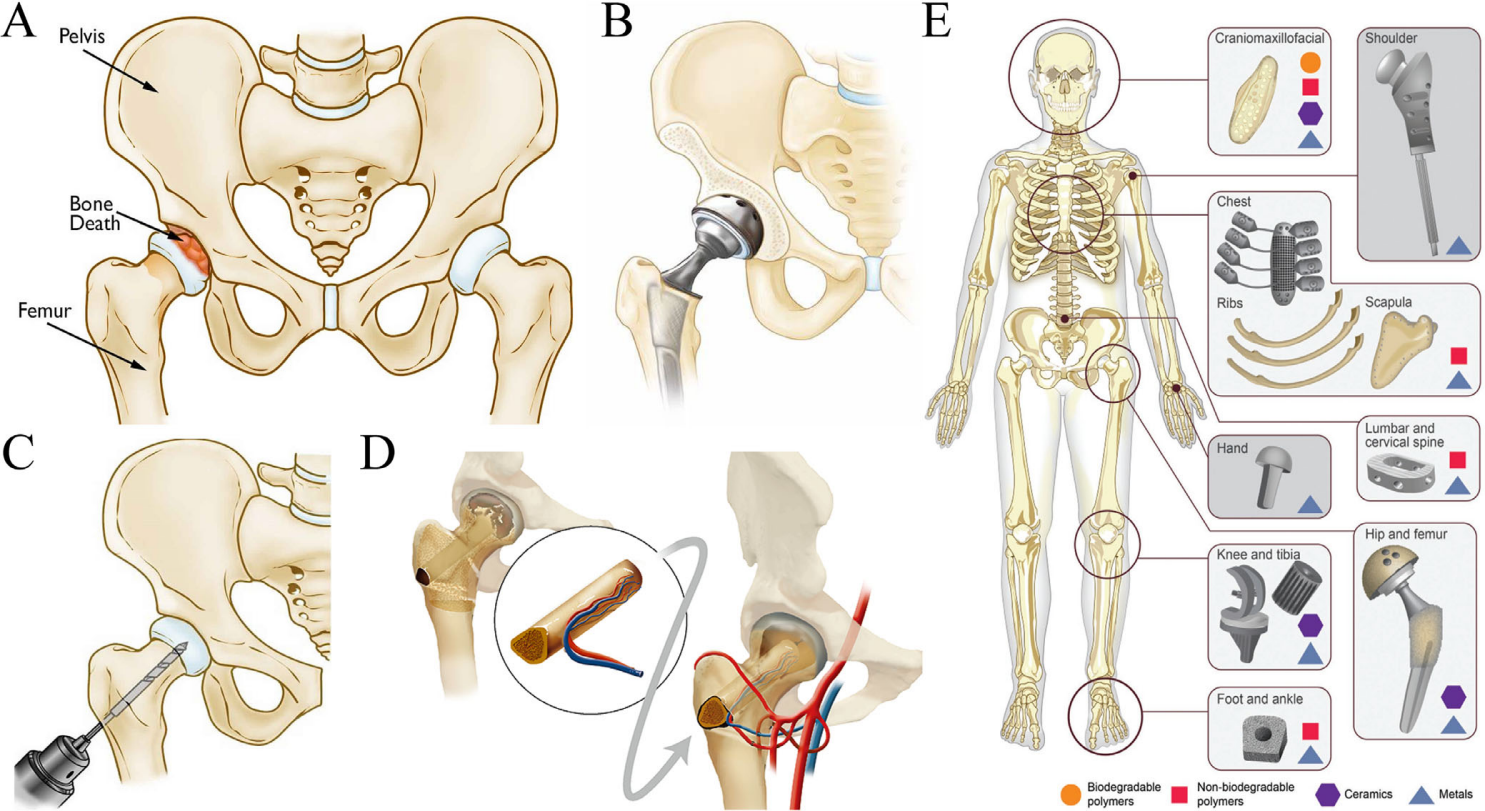
ONFH and current treatments. (A) ONFH is caused by bone death of the femoral head.^[^ ^1^ ^]^ (B) Total hip arthroplasty can reconstruct the hip joint function but may need to be revised in young patients.^[^ ^14^ ^]^ (C) Core decompression can alleviate the intraosseous pressure of the femoral head but cannot provide sufficient subchondral support or induce new bone formation.^[^ ^1^ ^]^ (D) Vascularized bone grafting can fill the tunnel drilled in core decompression to provide subchondral support and induce bone regeneration, but may bring some undesired complications.^[^ ^24^ ^]^ (E) Biomaterials are introduced to repair bone defects including ONFH. Reproduced with permission.^[^ ^25^ ^]^ Copyright 2017, John Wiley and Sons.

Osteonecrosis of the femoral head (ONFH) is a progressive disease that principally occurs in patients aged 30 to 50, in which osteocytes in the femoral head die due to hypoxia and ischemia (Figure 1A).^[^ ^1^ ^]^ If left untreated, ONFH can lead to subchondral bone collapse and hip joint dysfunction, which causes great pain and harm and thus seriously affect the quality of life.^[^ ^2^ ^]^ Although the etiology of ONFH remains unclear, trauma, alcohol abuse, steroid intake, heavy smoking, obesity, and basic autoimmune diseases are generally considered as the main pathogenic factors.^[^ ^3^, ^4^, ^5^, ^6^ ^]^ With the above pathogenic factors, the morbidity of ONFH has greatly increased in the 21st century, and more than 8 million people in China and 300,000 to 600,000 people in the United States suffer from ONFH.^[^ ^7^, ^8^ ^]^ With such a large patient population and a high disability rate, ONFH brings a tremendous economic burden to society.^[^ ^5^, ^8^, ^9^ ^]^

Currently, treatments of ONFH include conservative treatments and surgical treatments. Conservative treatments are only applicable to very early‐stage patients and cannot prevent ONFH deterioration.^[^ ^10^, ^11^ ^]^ Eighty percent of patients will further transform into femoral head collapse within 4 years without surgical intervention.^[^ ^12^ ^]^ Thus, surgeries are necessary for ONFH treatment, including femoral head‐preserving surgeries and total hip arthroplasty.^[^ ^13^ ^]^ Total hip arthroplasty can reconstruct hip joint function, but ONFH patients might have to receive multiple total hip arthroplasties due to prosthesis wear, inadequate fixation or osteoporotic bone (Figure 1B).^[^ ^14^, ^15^, ^16^ ^]^ Femoral head‐preserving surgery such as core decompression can alleviate the high intraosseous pressure and ischemia of the femoral head but cannot provide subchondral support or induce new bone and vascularity formation (Figure 1C).^[^ ^1^, ^17^, ^18^, ^19^, ^20^ ^]^ Combining nonvascularized or vascularized bone grafting with core decompression is an alternative approach to make up for these shortcomings.^[^ ^21^, ^22^, ^23^ ^]^ For example, the involvement of autogenous bone grafting (vascularized and nonvascularized) prolongs the surgery time and can cause complications such as hematoma, infection, and chronic pain (Figure 1D).^[^ ^24^ ^]^ For a long time, surgeons have been searching for alternatives to bone grafting to reduce surgery complications and improve the prognosis of ONFH. With a promising therapeutic perspective, bone tissue engineering has been increasingly applied in treating ONFH.

**FIGURE 2:**
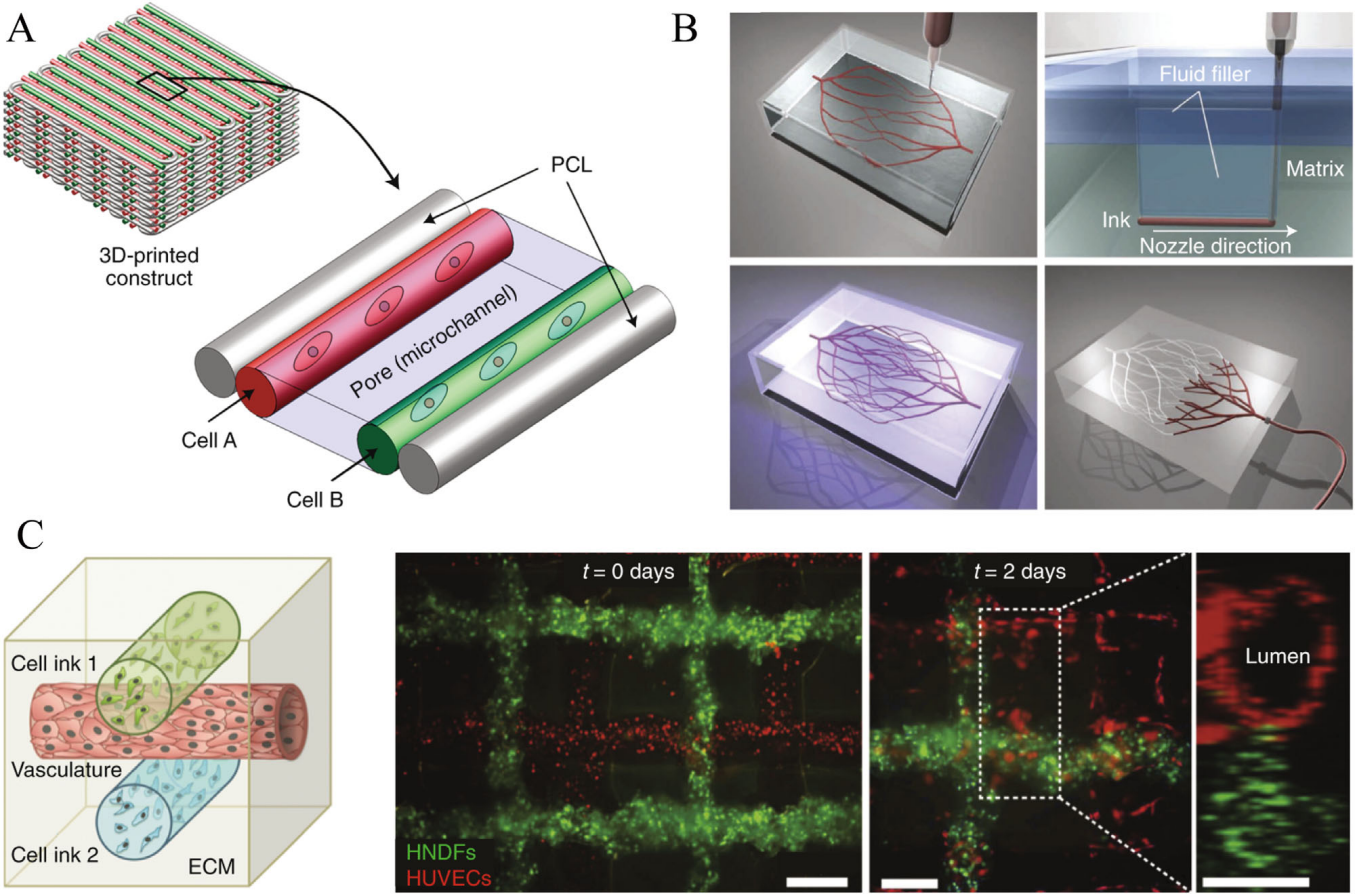
3D printing endows scaffold with desirable properties. (A) A 3D‐printed structure composed of cell‐landed hydrogels, supporting PCL, and intermediate microchannel for mimicking the anatomy and function of natural blood vessel. (B) The voids in hydrogel induced by the process of 3D printing of vascular architecture are filled with photocrosslinkable liquid and the microvascular channels are generated through subsequent photopolymerization and vacuum draw. (C) Hierarchical vascularity formation by 3D bioprinting using cells, vasculature, and extracellular matrix inks. Reproduced with permission.^[^ ^29^ ^]^ Copyright 2019, Springer Nature.

Bone tissue engineering combats controversial treatments by introducing biomaterials, stem cells, and bioactive factors to bone defects areas, of which biomaterial‐based scaffolds play an essential role not only in mimicking extracellular matrix but also in acting as a delivery system for bioactive cells and molecules (Figure 1E).^[^ ^25^, ^26^, ^27^ ^]^ Many factors should be considered when selecting and designing biomaterials for treating ONFH. First, the biomaterial must have satisfactory biocompatibility and osteogenic properties, which play an important role in reconstructing the necrotic femoral head. Second, the biomaterial needs to possess good mechanical properties and be space filling to provide sufficient and uniform subchondral support. Third, as the pathological basis of ONFH lies in the ischemia of the femoral head, so angiogenesis property is essential to reconstructing the blood supply for the necrotic femoral head.^[^ ^28^ ^]^ Finally, an unbiodegradable implant necessitates a second surgery to remove it, so biodegradable materials are preferred. Additive manufacturing (3D printing) is a promising fabrication technique and attracts more and more attention in customizing characteristics of scaffolds (Figure 2).^[^ ^29^ ^]^ Apart from bone‐repairing biomaterials, stem cell and bioactive molecule therapies have also been developed to dictate the adaptive and responsive properties of scaffolds.^[^ ^30^ ^]^

In this contribution, we present a comprehensive review to summarize the works in bone tissue engineering for treating ONFH. We first introduce the latest updated definition, classification, etiology, diagnosis, and treatments of ONFH. The development and cutting edge of various bone‐repairing biomaterials, including bioceramics, natural polymers, synthetic polymers, and metals, to treat ONFH is then described. After that, we discuss the development of biomaterials, including stem cells and bioactive molecules, for refined regenerative therapies of ONFH. This review captures both the latest research advances from the laboratory and clinical developments of bone tissue engineering for ONFH treatment. All the discussed clinical trials exploring the biomaterial or biological augmentation in ONFH treatment are summarized in Table 1. The causes of the success or failure of translated materials in clinical practice are also discussed to provide inspirations for subsequent material design. Finally, we conclude this review by providing some personal insights on the current challenges of these therapeutic strategies in the clinic and future development of bone tissue engineering for ONFH treatment.

**TABLE 1:** Clinical trials exploring the biomaterial or biological augmentation in ONFH treatment

Reference	Therapeutic strategy	No. of cases/No. of control subjects	Control	Patient age (Mean)(years)	Follow‐up	Stage system, No. of cases/No. of control subjects	Image evaluation	Clinical outcome	Level of evidence
Sun *et al.* ^[^ ^127^ ^]^ 2008	Nano‐hydroxyapatite/collagen and autogenous bone	26 patients (35 hips)	NA	34	2–7 years	ARCO, 6 stage IIB, 16 stage IIC, 9 stage IIIA, 3 stage IIIB, and 1 stage IIIC	X‐ray, CT	HHS	IV
Yang *et al.* ^[^ ^128^ ^]^ 2013	Nano‐hydroxyapatite/polyamide 66 and porous bioglass bone graft	29 patients (38 hips)/35 patients (46 hips)	Autologous cancellous bone graft	36	2 years	Steinberg, 3 stage IB, 4 stage IC, 8 stage IIA, 10 stage IIB, 8 stage IIC, 5 stage IIIA/3 IB, 5 stage IC, 9 stage IIA, 12 stage IIB, 11 stage IIC, 6 stage IIIA	X‐ray, MRI	HHS, VAS	III
Yamasaki *et al.* ^[^ ^130^ ^]^ 2010	Bone‐marrow‐derived mononuclear cells and interconnected porous calcium hydroxyapatite	22 patients (30 hips)/ 8 patients (9 hips)	Interconnected porous calcium hydroxyapatite	41	29 months	Japanese Orthopaedic Association, 2 stage I, 25 stage II, 3 stage III/ 9 stage II	X‐ray, CT, MRI	Merle d'Aubigné and Poste system, VAS	IV
Aoyama *et al.* ^[^ ^143^ ^]^ 2014	Autologous BMSCs and β‐TCP and vascularized bone grafts	10 patients (10 hips)	NA	32	1 year	SDIC, all stage 3	X‐ray, CT, MRI	Japan Orthopaedic Association score	IV
Hernandez *et al.* ^[^ ^140^ ^]^ 2019	Autologous bone marrow concentrate and β‐TCP	10 patients (10 hips)	NA	38	68.9 months	ACRO, 6 stage I, 4 stage II	X‐ray, MRI	HHS	IV
Kawate *et al.* ^[^ ^145^ ^]^ 2006	Autologous BMSCs and β‐TCP and free vascularized fibula	3 patients (3 hips)	NA	28	34 months	Steinberg, all stage 4	X‐ray, MRI	NA	IV
Lu *et al.* ^[^ ^147^ ^]^ 2019	Angioconductive bioceramic rod implant	200 patients (232 hips)	NA	42	22.7 months	ARCO, 150 stage II, 80 stage III	X‐ray	HHS	IV
Lu *et al.* ^[^ ^148^ ^]^ 2018	Angioconductive bioceramic rod implant	62 patients (72 hips)	NA	44	26.74 months	ARCO, 43 stage II, 29 stage III	X‐ray, MRI	HHS	IV
Huang *et al.* ^[^ ^151^ ^]^ 2008	Calcium phosphate cement/Danshen	48 patients (54 hips)	NA	39	42.5 months	ARCO, 9 stage I, 31 stage II, 14 stage III	X‐ray	HHS	IV
Yang *et al.* ^[^ ^182^ ^]^ 2010	DBM‐loaded allograft threaded cage	54 patients (56 hips)/22 patients (22 hips)	Core decompression	38	36–78 months	Steinberg, 26 stage I, 3 stage IIA, 19 stage IIB, 8 stage IIIA/18 stage IIA, 4 stage IIB	X‐ray	HHS	III
Helbig *et al.* ^[^ ^183^ ^]^ 2012	DBM	14 patients (18 hips)	NA	40	9 years	ARCO, 2 stage I, 16 stage II,	X‐ray, MRI	Merle d'Aubigné‐score	IV
Zhao *et al.* ^[^ ^276^ ^]^ 2016	Vascularized bone grafting and magnesium screw	23 patients (23 hips)/ 25 patients (25 hips)	Vascularized bone grafting	32	1 years	ARCO, 13 stage I, 10 stage II/13 stage I, 12 stage II	X‐ray, CT	HHS	II
Tsao *et al.* ^[^ ^286^ ^]^ 2005	Porous tantalum rod	98 patients (113 hips)	NA	43	4 years	Steinberg, 17 stage I and 96 stage II	X‐ray	HHS	IV
Veillette *et al.* ^[^ ^287^ ^]^ 2006	Porous tantalum rod	54 patients (60 hips)	NA	35	2 years	Steinberg, 1 stage I, 49 stage II, 8 stage III	X‐ray	HHS	IV
Liu *et al.* ^[^ ^295^ ^]^ 2014	Porous tantalum rod	149 patients (168 hips)	NA	33	5 years	ARCO, 79 stage II, 89 stage III	X‐ray, CT, MRI	HHS	IV
Zhao *et al.* ^[^ ^296^ ^]^ 2015	BMSCs and Tantalum rod and vascularized iliac graft	24 patients (31 hips)	NA	33	64.35 months	ARCO, 19 stage III, 12 stage IV	X‐ray	HHS	IV
Floerkemeier *et al.* ^[^ ^89^ ^]^ 2011	Tantalum rod	19 patients (23 hips)	NA	43	5 years	ACRO, 1 stage I, 22 stage II	MRI	HHS	IV
Chen *et al.* ^[^ ^327^ ^]^ 2019	Titanium metal trabecular bone reconstruction system	29 patients (32 hips)/24 patients (31 hips)	Vascularized fibular graft	39	7 years	ACRO, 3 stage IIA, 5 stage IIB, 9 stage IIC, 14 stage IIIA, 1 stage IIIB/4 stage IIA, 4 stage IIB, 10 stage IIC, 11 stage IIIA, and 2 stage IIIB	X‐ray, MRI	HHS, VAS	III
Zhang *et al.* ^[^ ^319^ ^]^ 2018	Titanium metal trabecular bone reconstruction system	30 patients (30 hips)	NA	Not available	2 years	ACRO, all stage II	X‐ray	HHS, VAS	IV
Yu *et al.* ^[^ ^333^ ^]^	Umbrella‐shaped, memory alloy	10 patients (18 hips)	NA	Not available	4 to 19 months	Ficat, 10 stage II, 6 stage III, 2 stage IV	X‐ray	HHS	IV
Zhao *et al.* ^[^ ^352^ ^]^ 2012	Core decompression & BMSC implantation	50 patients (53 hips)/50 patients (51 hips)	Core decompression	33	60 months	ACRO, 3 stage IC, 15 stage IIA, 23 stage IIB, 10 stage IIC/2 stage IC, 15 stage IIA, 22 stage IIB, 12 stage IIC	X‐ray	HHS	II
Hernigou *et al.* ^[^ ^359^ ^]^ 2002	Autologous bone marrow contained graft	116 patients (189 hips)	NA	31	5 to 10 years	Steinberg, 59 stage I, 86 stage II, 12 stage III, 32 stage IV	X‐ray	HHS	IV
Liu *et al.* ^[^ ^360^ ^]^ 2018	Autologous bone marrow mononuclear cells graft	148 patients (192 hips)	NA	38	5 years	CJFH, 27 C/M, 90 L1, 15 L2, 60 L3	X‐ray, MRI	HHS	IV

Abbreviations: ARCO, The Association Research Circulation Osseous system; HHS, Harris Hip Score; BMSCs, bone mesenchymal stem cells; SDIC, Specific Disease Investigation Committee; DBM, demineralized bone matrix; CJFH, China‐Japan Friendship Hospital typing system.

## 2 OSTEONECROSIS OF THE FEMORAL HEAD (ONFH)

### 2.1 Definition, etiology, and pathology of ONFH

ONFH, also called avascular necrosis of the femoral head, is the death of bone tissue of femoral head due to the interruption of the blood supply.^[^ ^2^, ^31^, ^32^ ^]^ ONFH can be divided into traumatic ONFH and nontraumatic ONFH according to etiology. Traumatic ONFH is associated with femoral neck fracture and other hip‐related trauma,^[^ ^33^ ^]^ while nontraumatic ONFH was significantly related to steroid intake, alcohol abuse, hematological diseases, and gene variation.^[^ ^34^, ^35^ ^]^

**FIGURE 3:**
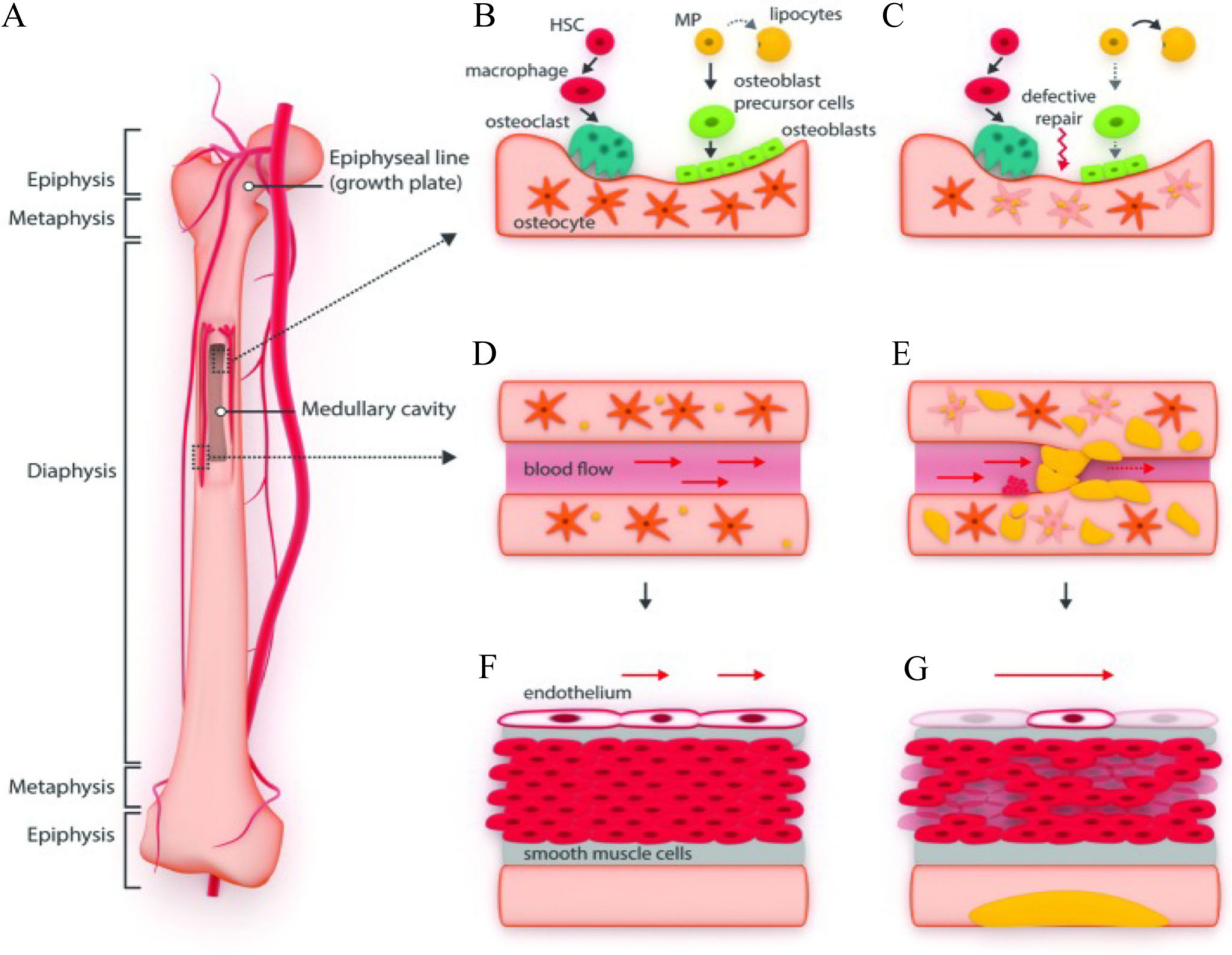
Schematic illustration of the pathogenesis mechanism of osteonecrosis. (A) Blood supply of femur and femoral head. (B,C) Bone homeostasis was regulated by endochondral bone formation and bone modeling. The activated osteoclasts, inhibited osteoblasts, and accumulated adipocytes account for the development of ONFH. (D,E) Blood supply of bone was obstructed by the hypertrophy, proliferation, and accumulation of adipocytes. (F,G) Direct endothelial and smooth muscle cell damage of arteries and veins reduce bone perfusion. Reproduced under the terms of the CC BY‐NC license.^[^ ^36^ ^]^ Copyright 2016, Kunstreich *et al.* HSC, hematopoietic stem cell; MP, mesenchymal progenitor cell.

Even though the underlying mechanism remains to be elucidated, the direct causes of ONFH can be concluded as mechanical vascular interruption, intravascular occlusion, and extravascular compression (Figure 3).^[^ ^36^, ^37^, ^38^ ^]^ Specifically, trauma‐caused ONFH is simply characterized by a mechanically disrupted vascular supply of the femoral head (mainly medial circumflex femoral). In contrast, nontraumatic ONFH involves complicated physiopathologic processes.^[^ ^39^ ^]^ Corticosteroid intake and alcohol abuse were reported to induce osteocyte and osteoblast death and activate the osteoclast by reducing the expression of osteogenic factors.^[^ ^40^, ^41^, ^42^, ^43^, ^44^ ^]^ Moreover, excessive corticosteroid or alcohol intake can promote the adipogenesis activity of bone mesenchymal stem cells (BMSCs), and the accumulated adipose tissue leads to hypertension of the bone marrow cavity, which further aggravates venous stasis and arterial blockage.^[^ ^42^, ^45^, ^46^, ^47^, ^48^ ^]^ Inhibited angiogenesis and promoted hypercoagulable state also contribute to the progression of corticosteroid and alcohol‐induced ONFH.^[^ ^49^, ^50^, ^51^, ^52^, ^53^ ^]^ Many hematological system diseases can also lead to ONFH. For example, abnormal or excessive erythrocytes in sickle cell disease or polycythemia vera can cause intravascular occlusion and ischemia of the femoral head.^[^ ^54^, ^55^, ^56^, ^57^, ^58^ ^]^ Intravascular occlusion caused by the imbalance between the coagulation system and fibrinolysis system can also lead to ONFH, as seen in hemophilia and hypercoagulable states‐related diseases.^[^ ^59^ ^]^ Patients with some autoimmune diseases need to take corticosteroid for a long period, as seen in systemic lupus erythematosus, which makes them susceptible to ONFH. Notably, patients with specific gene mutation (e.g., Type II collagen gene [COL2A1]) have a significantly higher tendency to ONFH, and people exposed to the same predisposing factors or medical comorbidities also present a different susceptibility to ONFH, indicating genetic variations play an important role in the occurrence of ONFH.^[^ ^60^ ^]^

Concerning pathology, necrotic femoral head presents a wedge‐shaped yellowish opaque appearance, discontinuities of bone trabecular and granulation tissue replacing the cystic lesions that caused by bone resorption can also be observed. HE staining features included disordered bone structure, bone marrow necrosis, and bone lacunae emptiness. Revascularization and neo‐fibrous tissue can also be seen in the margin of the lesion.^[^ ^61^, ^62^ ^]^

### 2.2 Diagnosis of ONFH

Early diagnosis plays a vital role in improving the prognosis of ONFH. Magnetic Resonance Imaging (MRI) and X‐ray imaging are the most commonly used imaging examinations for ONFH diagnosis.^[^ ^63^ ^]^ In MRI, ONFH presents a low‐intensity signal area on T1‐weighted and a high‐intensity signal area on T2‐weighted images. The sensitivity of MRI for ONFH has reached 100% and was considered the gold standard way to diagnose ONFH.^[^ ^64^ ^]^ For X‐ray imaging, the crescent sign and collapse are specific manifestations of ONFH, but very early‐stage ONFH is often not detectable. Despite new evidence demonstrated computed tomography (CT) has no longer be superior to the advanced MRI, it is sensitive to subchondral fractures and can serve as a supplementary diagnostic method.^[^ ^65^, ^66^ ^]^

After diagnosis, the accurate clinical stage of ONFH should be confirmed since it guides the subsequent treatment. Many systems have been proposed for defining and classifying ONFH stage, such as The Association Research Circulation Osseous (ARCO) system,^[^ ^67^ ^]^ Ficat and Arlet,^[^ ^68^ ^]^ Japanese Orthopaedic Association,^[^ ^69^ ^]^ and Steinberg/University of Pennsylvania systems.^[^ ^70^ ^]^ These systems define and classify the ONFH stages by accessing femoral head depression and collapse degree, lesion size and location, and acetabulum involvement. However, no consensus exists regarding the best system since they all bear limited interobserver and intraobserver reliability.^[^ ^71^, ^72^, ^73^ ^]^ The ARCO system that is typically based on the imaging manifestations and the depth of femoral head depression has been recently revised and aims to become the uniform classification tool by merging other classification systems (Table 2). However, more studies are needed to verify its practicability and reproducibility.^[^ ^67^ ^]^

**TABLE 2:** The 2019 revised ARCO classification system for osteonecrosis of the femoral head

ARCO Classification System for osteonecrosis of the femoral head	
Stage	Description
I	Normal radiograph and abnormal MRI findings
II	No crescent sign, radiographic evidence of sclerosis, osteolysis, or focal osteoporosis
III	Subchondral fracture, fracture in the necrotic portion, and/or flattening of the femoral head on radiograph or CT scan
IIIA	Femoral head depression of < 2 mm
IIIB	Femoral head depression of > 2 mm
IV	Evidence of osteoarthritis, joint space narrowing, and degenerative acetabular change

ARCO, Association Research Circulation Osseous; MRI, magnetic resonance imaging; CT, computed tomography.

*Source*: Adapted with permission.^[^ ^67^ ^]^ Copyright 2020, Elsevier.

### 2.3 Current treatments for ONFH

#### 2.3.1 Nonoperative therapies

Nonoperative therapies such as physical and drug therapies were used to alleviate the clinical symptoms of very early‐stage ONFH patients.^[^ ^11^, ^32^ ^]^ Physical therapy includes restricted weight‐bearing, acupuncture therapy, hyperbaric oxygen therapy, and extracorporeal shock wave therapy, while bisphosphonates, anti‐coagulants, vasodilators, statins, and traditional Chinese medicine are considered as effective drugs to relieve the clinical symptoms of ONFH patients.^[^ ^74^, ^75^, ^76^, ^77^, ^78^, ^79^, ^80^, ^81^, ^82^, ^83^ ^]^ However, the therapeutic effect of nonoperative therapies in halting ONFH progression is not encouraging.^[^ ^32^ ^]^ It is reported that more than 80% of the patients without surgical interventions will develop to femoral head collapse and require total hip arthroplasty (THA).^[^ ^12^ ^]^ Thus, for patients attempting to preserve the native joint, nonoperative therapy is not an appropriate option even in their early stages.

#### 2.3.2 Operative therapy

Core decompression is a classic femoral head‐preserving surgery used in the clinic for more than 50 years,^[^ ^84^ ^]^ which employs trochar/drill with a diameter ranging from 3 to 11 mm to drill one or multiple channel(s) through the necrotic area to reduce intraosseous pressure.^[^ ^11^, ^85^, ^86^, ^87^ ^]^ Despite the fact that favorable outcomes have been observed using core decompression to treat early‐stage ONFH, it should not be applied in late‐stage ONFH patients. It is reported that core decompression has a low failure rate of 14% to 25% for small lesions but a much high failure rate of 42% to 84% for large lesions.^[^ ^88^ ^]^ To improve the outcome of present therapies, bioactive materials and stem cells have been incorporated and achieved encouraging outcomes.^[^ ^89^, ^90^, ^91^ ^]^

Autologous bone grafting has long time been an ancillary procedure for core decompression in treating ONFH,^[^ ^84^ ^]^ which is often harvested from the fibula, iliac crest, and greater trochanter.^[^ ^92^, ^93^, ^94^, ^95^, ^96^ ^]^ Nonvascularized bone grafting can provide sufficient subchondral support and induce new bone formation, which allows remodeling of necrotic femoral head.^[^ ^23^, ^97^, ^98^ ^]^ Vascularized bone grafting can also rebuild the blood supply of the necrotic lesion to supply oxygen and remove metabolites.^[^ ^99^, ^100^, ^101^ ^]^ Nonetheless, the involvement of autologous bone grafting will prolong the surgery and postoperative rehabilitation time. Donor site complications such as hematoma, infection, and chronic pain also extremely limit its clinical application.^[^ ^102^, ^103^, ^104^ ^]^

THA can reconstruct hip joint function and is considered one of the most successful orthopedic surgeries.^[^ ^105^, ^106^ ^]^ For advanced‐stage ONFH, especially when the femoral head has collapsed or the acetabulum was implicated, THA was considered to be necessary.^[^ ^2^, ^107^, ^108^, ^109^, ^110^, ^111^, ^112^ ^]^ However, for younger patients (majority of ONFH patients), the prosthesis loosening and wear may happen with the prolonged use of artificial joints, which makes them face multiple surgeries during their lifetime and brings them huge psychological and economic burdens.^[^ ^113^ ^]^ Thus, protecting the native hip joint and delaying or preventing THA are the priorities for younger ONFH patients.

## 3 BONE‐REPAIRING BIOMATERIALS FOR ONFH TREATMENT

Bone‐repairing biomaterials are an essential component of bone tissue engineering in treating ONFH. A series of biomaterials and their composites possessing various therapeutic properties have been exploited to delay or prevent femoral head collapse in ONFH treatment, which are often fabricated into a porous rod shape and be implanted into the necrotic lesion of the femoral head to provide subchondral support and induce new bone and vascularity regeneration. Bioceramics, polymers (natural or synthetic), and metals are the most widely exploited materials in ONFH treatment. Compared to the regenerative therapies that involve stem cells or bioactive molecules, isolated material‐based scaffold holds the advantage of possessing better biosafety and clinical translation prospects. In the following parts, we will summarize the works that employ bone‐repairing biomaterial to treat ONFH and propose some perspectives on material‐design for ONFH treatment.

### 3.1 Bioceramics

Bioceramics are a class of inorganic materials that have been widely explored in bone tissue engineering, of which calcium phosphates and bioactive glasses received the most attention. The bioactive ions released from bioceramics mainly included Ca^2+^, PO_4_^3−^, Si^4+^, Mg^2+^, and Sr^2+^, which were considered to have the potential to induce bone regeneration.^[^ ^23^ ^]^ The porous surface of bioceramics also enhances their osteogenesis property. With excellent biocompatibility and osteoinductivity, bioceramics have been regarded as promising candidates for treating ONFH,^[^ ^114^, ^115^, ^116^ ^]^ among which hydroxyapatite (HA), beta‐tricalcium phosphate (β‐TCP), and calcium sulfate/calcium phosphate composite (CaSO_4_/CaPO_4_) are the most common used materials in repairing ONFH.

#### 3.1.1 Hydroxyapatite (HA)

**FIGURE 4:**
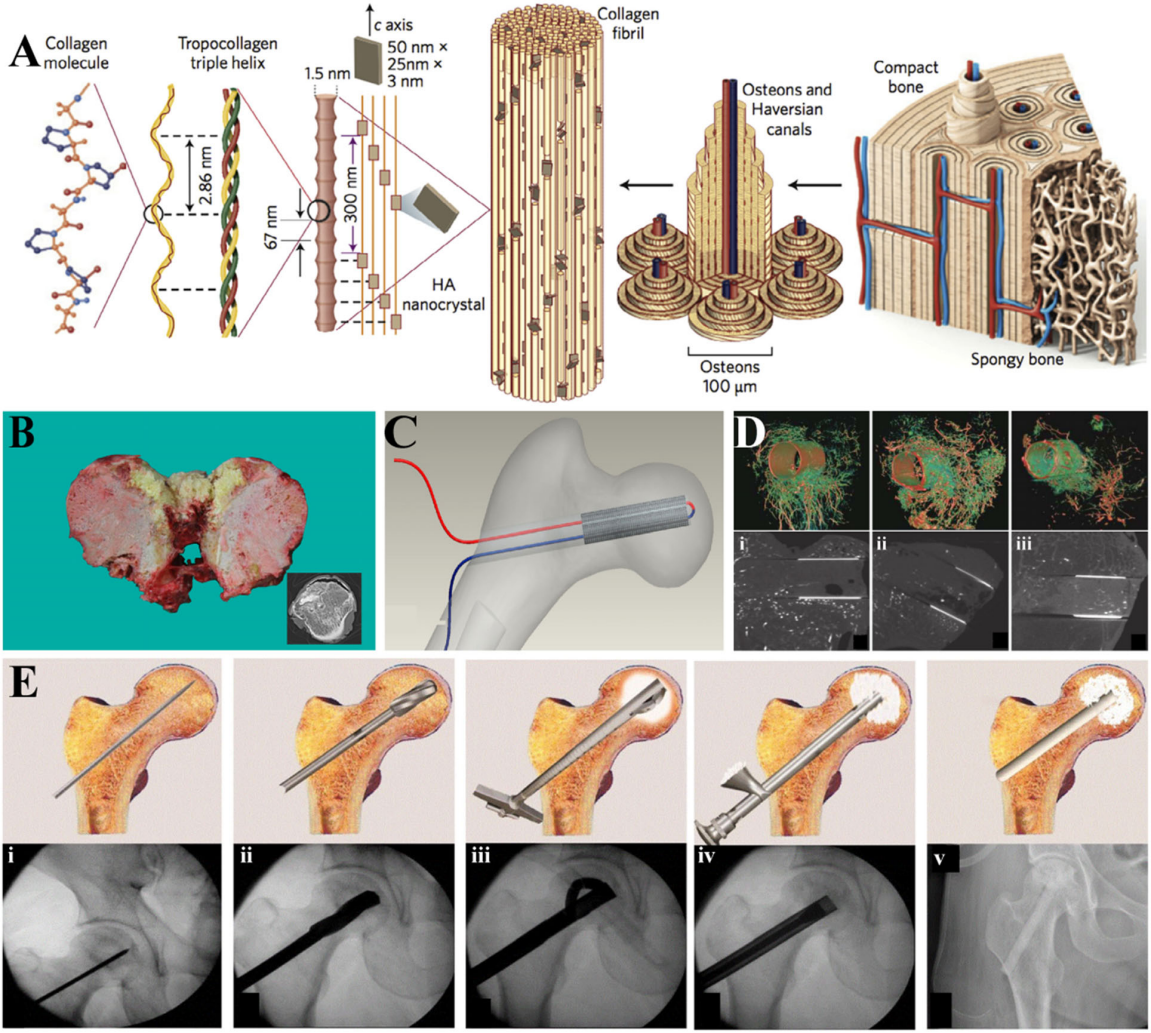
Ceramics used in repairing ONFH. (A) Bone is composed of type I collagen stiffened by crystals of HA, so HA is an ideal candidate for bone defect repair. Reproduced with permission.^[^ ^117^ ^]^ Copyright 2014, Springer Nature. (B) Pathological examination of the femoral head of failed case showed no new bone formation. Reproduced under the terms of the CC BY 4.0 license.^[^ ^144^ ^]^ Copyright 2021, Liu *et al.* (C) Schematic diagram of a novel porous implant with pre‐set channels to perform blood vessel implantation. Reproduced with permission.^[^ ^139^ ^]^ Copyright 2009, IOP Publishing. (D) Micro‐CT and corresponding three‐dimensional reconstruction images of the blood vessel around the β‐TCP‐based angio‐conductive bioceramic rod: (i) Four weeks; (ii) eight weeks; (iii) twelve weeks. Reproduced under the terms of the CC BY license.^[^ ^147^ ^]^ Copyright 2019, Lu *et al.* (E) Standard operation protocol of the angio‐conductive bioceramic rod implantation. Reproduced with permission.^[^ ^148^ ^]^ Copyright 2018, Springer Nature. CT, computed tomography.

As the inorganic component of natural bones, HA has a good biocompatibility and a satisfied hierarchical structure (Figure 4A).^[^ ^117^, ^118^, ^119^ ^]^ HA possesses satisfactory osteogenic properties, which were attributed to the Ca^2+^ and PO_4_^3−^ ions released from the HA. The released Ca^2+^ and PO_4_^3−^ can form an apatite layer on the surface of HA, which can induce bone regeneration directly and adsorb osteogenic proteins and factors to create an osteogenic microenvironment.^[^ ^120^ ^]^ Additionally, the porous surface topography of HA also creates favorable conditions for bone ingrowth. HA also possesses good mechanical properties and can provide sufficient support for the necrotic femoral head.^[^ ^26^, ^121^ ^]^ Combing good biocompatibility and favorable osteogenic and biomechanical properties, HA was considered as a promising material for ONFH treatment. The superiority of HA‐based composites in repairing ONFH has been reported. For example, Wang *et al.* ^[^ ^122^ ^]^ fabricated a self‐healing, injectable, and adhesive HA‐based composite with potent osteogenesis properties, which can significantly accelerate bone regeneration after being injected into the necrotic femoral head in an ONFH rabbit model. Gyawali *et al.* ^[^ ^123^ ^]^ developed an injectable composite composed of HA and biodegradable poly (ethylene glycol) maleate citrate (PEGMC). The composite possesses satisfied osteoconductivity and mechanical properties, which present a minimally invasive therapy for repairing ONFH. Moreover, HA can be a good carrier for drug delivery. Ma *et al.* ^[^ ^124^ ^]^ utilized HA to continuously release zoledronate (a bone absorption inhibitor) to the necrotic femoral head in rabbits. Histological and immunohistochemical results showed enhanced new bone formation and inhibited bone resorption in the HA/zoledronate groups 4 weeks after implantation, which verified the effective drug‐delivery ability of HA. Furthermore, HA‐based composites can also serve as stem cells and bioactive molecules delivery systems. Sun *et al.* ^[^ ^125^ ^]^ loaded bone marrow mesenchymal stem cells (BMMSCs) onto nano‐hydroxyapatite collagen (nHAC) and found that the scaffold has enhanced osteogenesis properties in repairing ONFH. Wang *et al.* ^[^ ^126^ ^]^ also loaded BMMSCs onto a composite composed of nano‐hydroxyapatite, collagen, and poly‐L‐lactic acid (nHAC/PLA). Increased neovascularization and bone regeneration in the necrotic femoral head were observed in treating ONFH of rabbits.

HA/nHA and their composites have also been performed in the clinic. Sun *et al.* ^[^ ^127^ ^]^ demonstrated that nHAC can repair and reconstruct the necrotic femoral head of early‐stage ONFH patients. They followed 26 patients (35 hips) for 2 to 7 years. Stable radiological manifestations were observed in 31 hips during the follow‐up, while the other 4 hips progressed from ARCO IIC to IIIA. Yang *et al.* ^[^ ^128^ ^]^ subsequently reported that nHA/polyamide 66 rod combining core decompression can delay ONFH progression. Sixty‐four patients (84 hips) with a mean age of 36.4 years were involved in the study and the mean follow‐up time was 2 years. The overall failure rate (defined as HHS < 80 or any further surgical procedure was needed) was 9/38 (23.68 %) in the nHA/polyamide 66 rod group and 24/46 (52.17 %) in the control group (treated with autologous cancellous bone graft). HA has also been co‐implanted with BMSCs to alleviate the symptoms of ONFH in the clinic.^[^ ^129^ ^]^ Yamasaki *et al.* ^[^ ^130^ ^]^ has investigated the therapeutic effect of the combination of BMSCs with interconnected porous calcium hydroxyapatite (IP‐CHA) in repairing ONFH. In this study, 22 ONFH patients (30 hips) received the IP‐CHA + BMSCs implantation and 8 patients (9 hips) received acellular scaffold implantation, and the mean follow‐up period was 29 months. Decreased necrotic volume and reduced collapse risk of the femoral head were observed in the BMSCs group. Three patients in BMSCs group (13.6%) and six patients in control group (75%) progressed to extensive collapse during the follow‐up.

#### 3.1.2 Beta‐tricalcium phosphate (β‐TCP)

Beta‐tricalcium phosphate (β‐TCP) is another bioceramic that possesses favorable biological and mechanical properties.^[^ ^131^, ^132^, ^133^ ^]^ Similar to HA, β‐TCP can also induce new bone formation and shares similar osteogenic mechanisms with HA. It is worth pointing out that β‐TCP can completely degrade in vivo, thus avoiding additional removal surgeries in ONFH treatment.^[^ ^134^, ^135^, ^136^, ^137^ ^]^ There are many reports about using β‐TCP to repair ONFH. For example, Li *et al.* ^[^ ^138^ ^]^ constructed a finite‐element model of ONFH with different necrotic volumes and collapse depth. Then a β‐TCP rod system was implanted into the ONFH finite‐element model. The results showed that the β‐TCP rod system could effectively prevent the femoral head collapse when the necrotic area was less than 15% or the collapse depth was less than 2 mm. However, lesions less than 15% were considered as small lesions, for which conservative treatments rather than surgery are recommended. Bian et al.^[^ ^139^, ^140^ ^]^ fabricated a novel porous implant with pre‐set channels with β‐TCP powder using the ceramic stereolithography technique, allowing the blood vessel implantation to rapidly revascularize the necrotic femoral head. Loading stem cells is another strategy to promote the osteogenic and angiogenic properties of β‐TCP‐based scaffolds. Different stem cell affinity peptides were identified and used to promote the adhesion, expansion, and proliferation of BMMSCs in β‐TCP scaffolds, which achieved favorable results in treating ONFH rabbits.^[^ ^141^, ^142^ ^]^

In clinic, the safety and efficacy of β‐TCP in repairing ONFH have been accessed. Aoyama *et al.* ^[^ ^143^ ^]^ combined β‐TCP with autologous BMMSCs and vascularized bone grafts to treat nine ONFH patients with collapsed femoral head (identified as stage III according to the Specific Disease Investigation Committee). After 24 months' follow‐up, seven patients remained at stage III, while the other two progressed to stage IV. Increased mean bone volume and ameliorated clinical symptoms were observed in these patients. However, as the follow‐up time prolonged, many failures were also reported. In a study using β‐TCP combining core decompression in repairing ONFH that involved 10 patients (10 hips) with a mean 68.9 months' follow‐up time, a high failure rate of 74.5% was reported (Figure 4B).^[^ ^144^, ^145^ ^]^ Kawate *et al.* ^[^ ^146^ ^]^ also reported a high failure rate of applying β‐TCP to treat stage IV (Steinberg) ONFH patients, which enrolled 3 patients with a mean follow‐up time of 34 months. The high failure rate was attributed to the ultrafast biodegradation rates of β‐TCP.^[^ ^145^ ^]^ If the biodegradation of β‐TCP is much faster than the bone remodeling, the femoral head collapse will occur. Moreover, β‐TCP was also criticized for its impaired osteogenesis and angiogenesis properties. Many efforts have been made to optimize the angiogenic property of β‐TCP scaffolds. A blood‐conductive β‐TCP rod was also designed and used to treat ONFH patients in two studies, which enrolled 262 patients (304 hips) with a mean follow‐up time of about 2 years. The implant was proved to conduct blood supply from the great trochanter and femoral neck to the necrotic femoral head, thus promoting ONFH healing (Figure 4C–E).^[^ ^139^, ^147^, ^148^ ^]^

#### 3.1.3 Other bioceramics

Calcium phosphate cement (CPC) with satisfying mechanical properties has been widely exploited in treating ONFH. It is suggested that filling CPC into the tunnel after core decompression can increase the subchondral stability of the femoral head. The slow degradation rate of CPC can prolong subchondral support, which benefits gradual bone regeneration and complete bony substitution.^[^ ^149^ ^]^ Chang *et al.* ^[^ ^150^ ^]^ fabricated a novel scaffold composed of CPC, poly (propylene fumarate), and ginsenoside Rg1. The CPC component could promote the mechanical properties and reduce the cytotoxicity of the scaffold. The addition of ginsenoside Rg1 endowed the scaffold with potent angiogenesis properties, which play an important role in reconstructing the microcirculation of the necrotic femoral head.

CPC was also been explored in clinic. For example, Jiang *et al.* ^[^ ^151^, ^152^ ^]^ combined Danshen (a traditional Chinese herb that can promote angiogenesis) with CPC to repair ONFH and achieve satisfactory results. In contrast, failures occurred in those applying CPC without the addition of angiogenic bioactive molecules. For example, Rijnen *et al.* ^[^ ^153^ ^]^ demonstrated that the combination of CPC with morselized cancellous bone could not prevent femoral head collapse. This combination showed inferior bony remodeling because of the inadequate revascularization of the necrotic femoral head.

Calcium sulfate/calcium phosphate composite (CaSO_4_/CaPO_4_) is an injectable and degradable bioceramic. The degradation of CaSO_4_/CaPO_4_ can be divided into a short period of abrupt degradation followed by a gradual dissolution.^[^ ^154^ ^]^ It was reported that CaSO_4_/CaPO_4_ can effectively alleviate the clinical symptoms and delay femoral head collapse in repairing ONFH.^[^ ^90^, ^155^ ^]^ Recently, a novel therapy combining core decompression with injectable CaSO_4_/CaPO_4_ achieved success in clinic in repairing ONFH.^[^ ^156^, ^157^, ^158^ ^]^ It is suggested that the therapy can promote femoral neck stability and accelerate bony rehabilitation. However, others argued that the implantation of CaSO_4_/CaPO_4_ can only improve the prognosis of alcohol‐induced ONFH, but not steroid‐induced ONFH, and has a high failure rate in treating ARCO stage IIC and IIIA ONFH.^[^ ^159^, ^160^ ^]^ Thus, CaSO_4_/CaPO_4_ may only be appropriate to repair early‐stage ONFH, and future studies are needed to confirm their long‐term therapeutic effect.

### 3.2 Natural polymers

Natural polymers are a class of polymeric biomaterials derived from a living source. These polymers possess excellent biocompatibility and negligible immunoreactivity, and thus they can be safely implanted into the human body. With high water content, natural polymers bear poor mechanical properties, so they are often combined with other tough materials to provide sufficient support for the subchondral bone of the femoral head when treating ONFH.^[^ ^161^ ^]^ Among natural polymers, collagen, gelatin hydrogel, and silk fibroin (SF) are the most commonly explored biomaterials in repairing ONFH.

#### 3.2.1 Collagen

**FIGURE 5:**
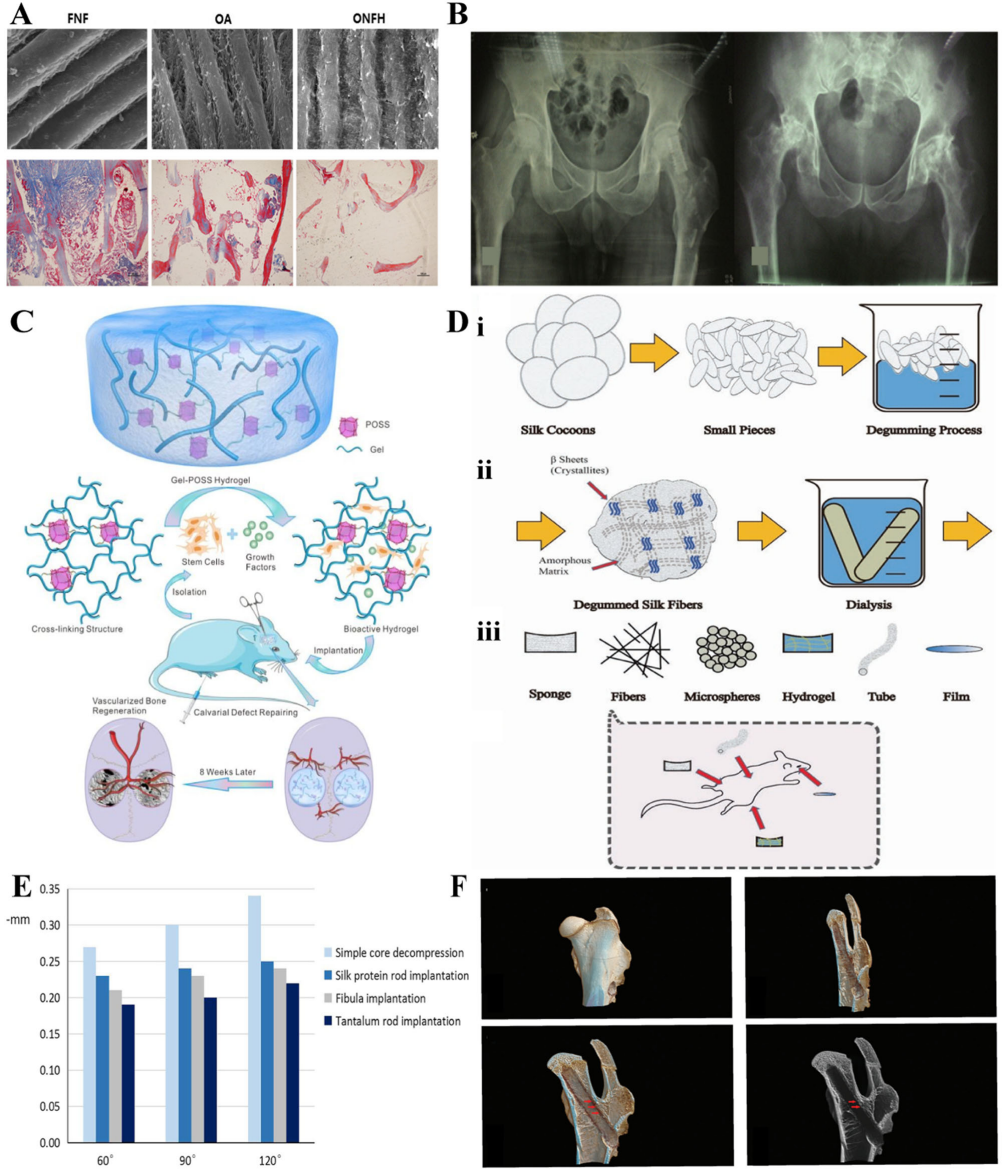
Promising natural polymers in repairing ONFH. (A) Scanning electron microscopic and histological images showed significantly decreased collagen in the femoral head of ONFH compared with femoral neck fracture (FNF) and osteoarthritis (OA). Reproduced under the terms of the CC BY 4.0 license.^[^ ^170^ ^]^ Copyright 2020, Wu *et al.* (B) A 39 years old male with bilateral ONFH received DBM‐filled allograft threaded cage insertion on the left side and no treatment on the right side (i); the DBM‐filled allograft threaded cage has been obviously absorbed and negligible progression was observed in the left hip while significant collapse occurred in the right hip (ii). Reproduced with permission.^[^ ^182^ ^]^ Copyright 2010, Elsevier. (C) Schematic diagram of the structure of a gelatin‐based hydrogel that can induce vascularized bone regeneration in vivo. Reproduced with permission.^[^ ^194^ ^]^ Copyright 2020, American Chemical Society. (D) Illustration of silk fibroin processing (i); schematic representation of different silk fibroin forms (ii); multiple applications of silk fibroin in tissue regeneration engineering (iii). Reproduced with permission.^[^ ^209^ ^]^ Copyright 2018, Springer Nature. (E) Less femoral head displacement was observed in the silk protein rod group compared with the simple core decompression group. Reproduced under the terms of the CC BY 4.0 license.^[^ ^214^ ^]^ Copyright 2019, Huang *et al.* (F) Reconstructed micro‐CT images show that the silk‐based scaffold can provide mechanical support for the necrotic femoral head and induce in situ new bone formation (red arrow). Reproduced with permission.^[^ ^215^ ^]^ Copyright 2020, Royal Society of Chemistry. POSS, polyhedral oligomeric silsesquioxane; gel, gelatin.

Collagen is the main polymeric component of natural bone, which acts as a scaffold for HA and bone cells and thus endows the bone with tensile strength.^[^ ^162^, ^163^ ^]^ Excellent osteogenesis and angiogenesis properties have been observed in collagen‐based scaffolds.^[^ ^164^, ^165^, ^166^, ^167^, ^168^ ^]^ It has been reported that collagen abnormality partly accounts for ONFH pathogenesis (Figure 5A).^[^ ^169^, ^170^ ^]^ COL2A1 mutation can also lead to defective hierarchical cartilage structures and abnormal bone homeostasis, contributing to the development of ONFH.^[^ ^60^, ^171^, ^172^, ^173^, ^174^, ^175^, ^176^, ^177^ ^]^ As a key factor in ONFH pathogenesis, collagen has attracted much attention and has been widely exploited in repairing ONFH. In practice, collagen was often combined with tough material to obtain good mechanical properties. For example, the collagen/hydroxyapatite composite and collagen/porous tantalum can both provide sufficient subchondral support for the necrotic femoral head and repair the cartilage defects in ONFH effectively.^[^ ^125^, ^126^, ^127^, ^178^ ^]^

Demineralized bone matrix (DBM) is another form of collagen derived from natural bones through demineralization and cell elimination. DBM contains abundant osteogenic proteins such as bone morphogenetic proteins (BMP) and shows excellent osteogenesis properties.^[^ ^179^ ^]^ Yang *et al.* ^[^ ^180^, ^181^, ^182^ ^]^ conducted a clinical trial and demonstrated that a DBM‐filled allograft threaded cage could delay or arrest the progression of ONFH, 54 patients (56 hips) enrolled in this study showed reduced pain and improved hip joint function with the clinical success rate of the scaffold reaching 91% with a minimum follow‐up period of 36 months (Figure 5B). In another study conducted by Helbig *et al.* ^[^ ^183^ ^]^ that enrolled 14 patients (18 hips) with a 2‐year follow‐up for imaging results and 9‐year follow‐up for clinical results, DBM combining cannulated bone screws were found to accelerate bone healing in early‐stage ONFH patients. However, it should be noted that DBM is not appropriate for repairing end‐stage ONFH or ONFH with large necrotic volume. Thus, further studies are encouraged to verify the long‐term therapeutic effect of DBM graft, especially when the necrotic volume of the femoral head is large.

#### 3.2.2 Gelatin hydrogel

Gelatin is the hydrolysis product of collagen and possesses excellent biocompatibility and biodegradability.^[^ ^184^, ^185^, ^186^ ^]^ The arginine‐glycine‐aspartic acid (RGD) and matrix metalloproteinase (MMP) amino acid sequences in gelatin make it superior in promoting cell adhesion, remodeling, and proliferation.^[^ ^187^, ^188^, ^189^ ^]^ Although the mechanical strength of gelatin is low because of its abundant water content, its compressive modulus can be finely tuned by combining other inorganic or organic components.^[^ ^190^, ^191^, ^192^, ^193^ ^]^ With tunable mechanical properties, progress has been achieved in the utilization of gelatin‐based scaffolds in bone tissue engineering (Figure 5C).^[^ ^194^, ^195^, ^196^, ^197^, ^198^, ^199^ ^]^ When used for repairing ONFH, gelatin was often served as a delivery system to deliver bioactive molecules and drugs. Li *et al.* ^[^ ^121^ ^]^ developed a scaffold composed of lithium, nHA, gelatin microsphere, and erythropoietin (Li‐nHA/GMs/rhEPO). in vivo studies demonstrated the erythropoietin released from the gelatin microsphere can promote new bone and vascularity formation in the necrotic femoral head of rabbits. Similarly, vascular endothelial growth factor (VEGF) was also loaded in a gelatin microsphere to endow the biomaterial with angiogenesis properties.^[^ ^200^ ^]^ In vitro studies found the VEGF can be continuously released from the gelatin microsphere for 30 days, which significantly promotes the neovascularization after the scaffold has been implanted into the necrotic femoral head of rabbits ONFH model. The injectable gelatin hydrogel can also control the release rate of biological factors to avoid the unwanted dissemination of these factors. For example, Phipps *et al.* ^[^ ^201^ ^]^ designed and fabricated a self‐assembling peptide hydrogel that can control the BMP‐2 release within the femoral head to avoid heterotopic ossification in the hip capsule, providing a benefit to ONFH patients suffering heterotopic ossification. In clinic, Kuroda *et al.* ^[^ ^202^ ^]^ impregnated gelatin hydrogel in recombinant human fibroblast growth factor‐2 (rhFGF‐2) before scaffold implantation. The local release of rhFGF‐2 continued for more than two weeks after implantation, which perfectly matched the degradation pattern of gelatin hydrogel.

#### 3.2.3 Silk fibroin (SF)

SF is another natural polymer derived from silkworms. The primary compositions and degradation products are glycine and alanine, which can be recycled to produce new proteins in vivo.^[^ ^203^, ^204^, ^205^ ^]^ The degradation rate of SF is adjustable to accommodate bone remodeling by manipulating its secondary structure.^[^ ^206^, ^207^, ^208^ ^]^ With great flexibility, SF has been manufactured into many forms, expanding its application (Figure 5D).^[^ ^209^ ^]^ Unlike other natural polymers, SF has outstanding mechanical properties, which can provide sufficient support for the necrotic femoral head.^[^ ^210^, ^211^, ^212^, ^213^ ^]^ Huang *et al.* ^[^ ^214^ ^]^ established a three‐dimensional (3D) finite element model of SF rod. The SF rod was implanted into the ONFH model to investigate its mechanical properties. The results indicated that the silk protein rod could reduce the collapse and surface stress at the weight‐bearing area of the necrotic femoral head (Figure 5E). Wang *et al.* ^[^ ^215^ ^]^ combined SF with hydroxypropyl methylcellulose (SF/HPMC) fabricating a scaffold with great mechanical properties. Satisfied osteoinductivity and sufficient mechanical support were observed after implanting the scaffold into the necrotic femoral head in rabbits (Figure 5F). Notably, the biodegradation rate of the scaffold can be finely regulated to accommodate the bone regeneration rate, thus realizing the bone‐scaffold replacement seamlessly. However, no clinical studies are identified using SF to treat ONFH, so further efforts are needed to translate SF into clinical practice.

### 3.3 Synthetic polymers

**FIGURE 6:**
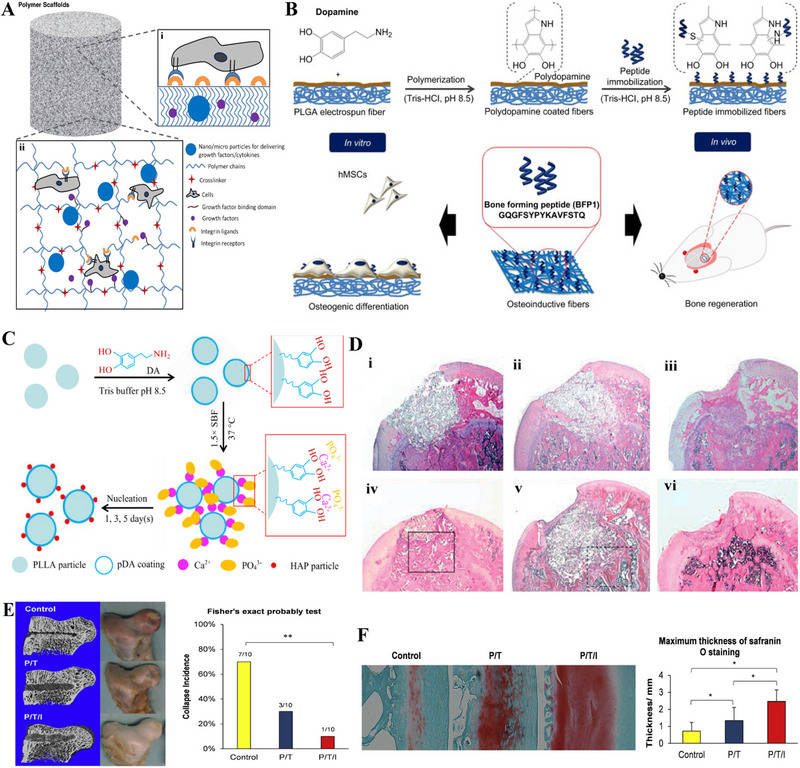
Promising synthetic polymers in repairing ONFH. (A) Strategies for bone regeneration using hydrogel‐based polymer implants. Reproduced with permission.^[^ ^217^ ^]^ Copyright 2015, Elsevier. (B) Schematic illustration of the surface modification of PLGA electrospun fibers by peptides (polydopamine and bone‐forming peptide) obtaining enhanced osteogenesis property. Reproduced under the terms of the CC BY license.^[^ ^218^ ^]^ Copyright 2014, Gentile *et al.* (C) pDA‐coated PLLA particles have favorable osteogenesis properties and can in situ generate hydroxyapatite particles on their surface. Reproduced with permission.^[^ ^118^ ^]^ Copyright 2020, American Chemical Society. (D) Postoperative H&E staining of femoral heads of Adv‐Cbfa1/OPLA‐transplanted rats (i,iv), uninfected OPLA‐transplanted rats (ii,v), and untreated defects (iii,vi) in week 1 (i–iii) and week 3 (iv–vi). Enhanced bone regeneration was observed in the Adv‐Cbfa1/OPLA‐transplanted group in week 3. Reproduced with permission.^[^ ^241^ ^]^ Copyright 2020, John Wiley and Sons. (E) Left and middle: Micro‐CT image and gross view of the scaffold‐transplanted/control femoral head taken 12 weeks post‐operation. Right: Fisher's exact probability test demonstrated that the femoral head in the P/T/I group bore significantly lower collapse incidence compared with the control and P/T group. Reproduced with permission.^[^ ^252^ ^]^ Copyright 2015, Elsevier. (F) Left: Safranin O and fast green staining of emu's femoral head articular cartilage. Right: The maximum thickness of cartilage matrix. Reproduced with permission.^[^ ^252^ ^]^ Copyright 2015, Elsevier. pDA, poly dopamine; H&E, hematoxylin‐eosin; Adv‐Cbfa1, Cbfa1 recombinant adenovirus; P/T, poly lactic‐*co*‐glycolic acid/tricalcium phosphate; P/T/I, poly lactic‐*co*‐glycolic acid/tricalcium phosphate/icaritin.

Synthetic polymers are artificial polymers that are normally synthesized from their precursors through chemical reactions. Compared with natural polymers, synthetic polymers possess better mechanical properties and chemical susceptibility.^[^ ^216^ ^]^ Despite synthetic polymers exhibitin better mechanical properties than natural polymers, there is no consensus whether they can provide sufficient support for the femoral head when used alone. Generally, synthetic polymers are often combined with bioceramics in treating ONFH. However, the biocompatibility and osteogenesis properties of synthetic polymers are relatively poor, so synthetic polymers are often combined with other biomaterials or biological therapies in bone tissue engineering (Figure 6A,B).^[^ ^217^, ^218^ ^]^ Currently, the most widely used synthetic polymers in repairing ONFH include poly (ε‐caprolactone) (PCL), polylactic acid (PLA)/poly (L‐lactide acid) (PLLA), and poly (lactic‐*co*‐glycolic acid) (PLGA). However, despite many studies demonstrating promising therapeutic effects of synthetic polymers in ONFH treatment, clinical translation has not been achieved yet, which suggests that further studies should pay more attention to the gaps between material‐design and clinical application.

#### 3.3.1 Poly (ε‐ caprolactone) (PCL)

PCL is a semi‐crystalline polymer with superior mechanical properties.^[^ ^219^ ^]^ The hydrophobic nature of PCL can suppress cell adhesion and proliferation, so PCL is often combined with other biomaterials such as bioceramics or natural polymers to acquire improved biocompatibility.^[^ ^220^, ^221^, ^222^ ^]^ Kawai *et al.* ^[^ ^223^ ^]^ combined PCL with β‐TCP to fabricate a scaffold with good biocompatibility and osteogenesis properties; the scaffold can provide sufficient subchondral support for the necrotic femoral head. Maruyama *et al.* ^[^ ^224^ ^]^ co‐implanted a PCL/β‐TCP scaffold with BMMCs into the necrotic femoral head of rabbit ONFH model, and found that the strategy can induce new bone formation and reduce the necrotic volume. Moreover, the combination of PCL with other synthetic polymers has also been explored. Zhu *et al.* ^[^ ^225^ ^]^ fabricated a PLLA/PLGA/PCL/BMP‐2 composite with sustainable BMP‐2 release ability. The combination of the scaffold with low‐intensity pulsed ultrasound (LIPUS) therapy can promote the healing process of steroid‐induced ONFH in rats, providing a potential therapeutic option for ONFH treatment.^[^ ^226^, ^227^, ^228^ ^]^

#### 3.3.2 Polylactic acid (PLA)

PLA is a commercially available material and has been extensively used in the medical field, including sutures, drug delivery systems, and tissue engineering.^[^ ^229^, ^230^, ^231^ ^]^ PLA has great mechanical properties and is considered as a promising candidate for bone repairing.^[^ ^232^, ^233^, ^234^, ^235^, ^236^ ^]^ The degradation rate of PLA in vivo is very slow but can be accelerated by introducing other biodegradable materials.^[^ ^237^, ^238^, ^239^ ^]^ Apart from the great mechanical property and adjustable degradation rate, PLA can be easily blended with other biomaterials to acquire desirable properties (Figure 6C).^[^ ^118^, ^229^, ^230^, ^240^ ^]^

Several studies have designed PLA‐based scaffolds to treat ONFH. For example, Sakai *et al.* ^[^ ^241^ ^]^ fabricated a 3D porous PLA scaffold and loaded the scaffold with adenoviral vectors carried core‐binding factor α1 (Cbfa1) genes. Infiltrated osteoblast and accelerated bone regeneration were observed in the necrotic femoral head three weeks after implantation (Figure 6D). Lu *et al.* ^[^ ^242^ ^]^ developed a novel scaffold composed of PLA, nHA, and recombinant human‐like collagen. Autogenous osteoblasts and endotheliocytes were loaded on the scaffold to repair ONFH in canine. Compared with the control group, enhanced bone and vascularity regeneration were observed in the scaffold‐implanted group. According to existing literature, PLA tends to serve as structural support for the necrotic femoral head, and thus it is advisable to combine PLA with other osteogenic and angiogenic biomaterials or regenerative therapies when used for repairing ONFH.

#### 3.3.3 Poly(lactic‐*co*‐glycolic) acid

PLGA is another important synthetic polymer formed by the polymerization of lactic acid and glycolic acid. PLGA possesses great biosafety and tunable degradation rate, attracting much attention in bone tissue engineering.^[^ ^243^, ^244^, ^245^ ^]^ However, the poor osteogenesis property and unsatisfied mechanical property of PLGA make it suboptimal for repairing load‐bearing bones.^[^ ^246^ ^]^ Therefore, PLGA has been combined with ceramics, natural polymers, and other synthetic polymers to compromise these drawbacks.^[^ ^247^, ^248^, ^249^, ^250^, ^251^ ^]^

The combination of PLGA with β‐TCP has been explored in repairing ONFH. For example, a PLGA/β‐TCP scaffold loading with icaritin was fabricated to treat a steroid‐associated ONFH model in emu (a large bipedal flightless bird). It turned out the scaffold can provide sufficient subchondral support and protect the femoral head from collapse. Enhanced osteogenesis and chondrogenesis and inhibited adipogenesis were also observed in the necrotic femoral head (Figure 6E,F).^[^ ^252^ ^]^ Zhang *et al.* ^[^ ^253^ ^]^ designed a novel scaffold composed of CPC and PLGA. The scaffold was loaded with BMP and VEGF and the loading efficiency reached 89.15% and 78.55%, respectively. The good biocompatibility, outstanding osteogenesis, and angiogenesis properties of the scaffold were verified In vitro. in vivo results also showed enhanced new bone and vascularity formation six weeks after scaffold implantation, providing a potential strategy for ONFH treatment.

## 4 METALS FOR ONFH TREATMENT

Metals are tough enough to provide sufficient subchondral support for the necrotic femoral head. However, the mechanical modulus of metals is too high and tends to cause a stress shielding effect, which would hinder bone reconstruction when used to repair bone defects. Recent advances in additive manufacturing and topology optimization technique bring unprecedented opportunities for modulating the mechanical properties of metals by adjusting their external and internal structures.^[^ ^254^ ^]^ Other obstacles hindering the application of metals include implant loosening and infection, which can be combated by optimizing surface modification techniques.^[^ ^255^ ^]^ Up to now, magnesium, tantalum, and titanium are the most widely explored metals in repairing ONFH.

### 4.1 Magnesium

**FIGURE 7:**
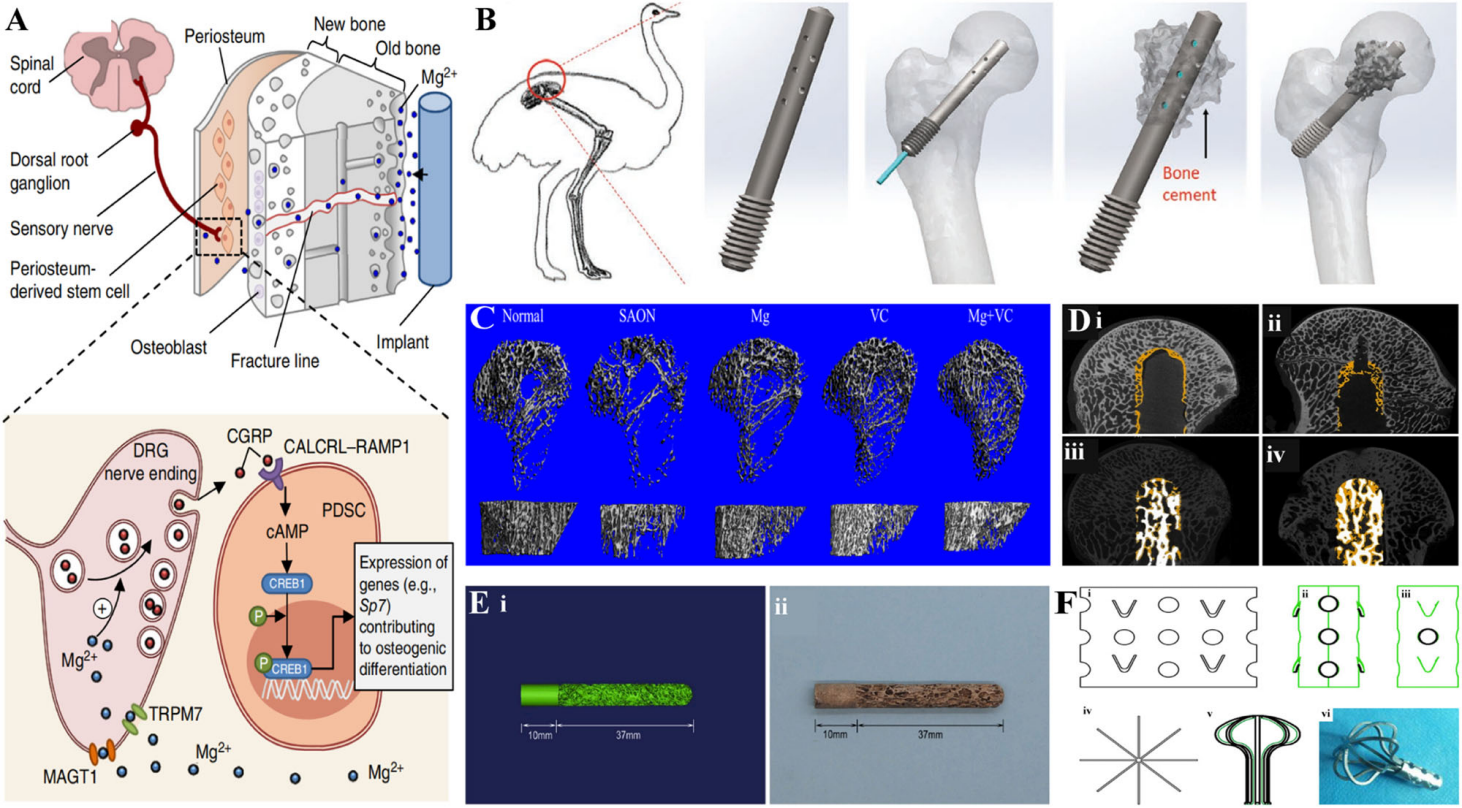
Promising metals in repairing ONFH. (A) Schematic diagram showing the released Mg^2+^ from the implant promote the osteogenic differentiation of periosteum‐derived stem cells (PDSCs) that regulated by the dorsal root ganglion (DRG) sensory neurons. Reproduced with permission.^[^ ^260^ ^]^ Copyright 2016, Springer Nature. (B) The bone cement can be injected through the holes in the implanted magnesium‐based screw to repair ONFH in emu. Reproduced under the terms of the CC BY license.^[^ ^263^ ^]^ Copyright 2020, Wang *et al.* (C) 3D micro‐CT images of proximal tibia show significantly enhanced trabecular bone density in the magnesium/vitamin C (VC) group 6 weeks after SAON induction. Reproduced with permission.^[^ ^275^ ^]^ Copyright 2020, Elsevier. (D) Micro‐CT images show the new bone growth (the yellow area) in the femoral head of the core decompression group (i,ii) and the 3D printed porous titanium alloy rod implantation group (iii,iv) 3 months (i,iii) and 6 months (ii,iv) postoperatively. Significantly enhanced bone regeneration was observed in the titanium rod group, and no radiolucent lines and fibrous tissue around the implanted rod were observed. Reproduced with permission.^[^ ^324^ ^]^ Copyright 2020, Elsevier. (E) Digital models of a biogenic trabecular rod and a 3D printed porous titanium alloy rod with excellent osteogenesis and osteointegration properties. Reproduced with permission.^[^ ^324^ ^]^ Copyright 2020, Elsevier. (F) Umbrella‐shaped femoral head support device. Reproduced with permission.^[^ ^333^ ^]^ Copyright 2013, Springer Nature. CGRB, calcitonin gene‐related polypeptide‐a; MAGT1, magnesium induces magnesium transporter 1; TRPM7, transient receptor potential cation channel, subfamily M, member 7; cAMP, cyclic adenosine monophosphate; CREB1, cAMP‐responsive element binding protein 1; VC, vitamin C.

Magnesium possesses outstanding biocompatibility and osteogenesis properties (Figure 7A).^[^ ^256^, ^257^, ^258^, ^259^, ^260^ ^]^ As a metal biomaterial, magnesium is strong enough to meet the mechanical requirements for repairing ONFH.^[^ ^261^ ^]^ Unlike other metals with much higher mechanical properties than natural bones that cause stress shielding effects, the mechanical property of magnesium is similar to that of natural cortical bones.^[^ ^262^, ^263^, ^264^, ^265^ ^]^ Biodegradability is another fantastic property of magnesium, which avoids extra removal surgery. The degradation byproducts of magnesium are magnesium hydroxide and hydrogen gas, which can be metabolized in vivo and cause no adverse effects.^[^ ^266^, ^267^, ^268^ ^]^ With desirable properties, magnesium has been widely used in bone tissue engineering for ONFH treatment (Figure 7B).^[^ ^263^, ^269^, ^270^, ^271^, ^272^, ^273^, ^274^, ^275^, ^276^ ^]^

He *et al.* ^[^ ^272^ ^]^ found magnesium‐substituted wollastonite can promote the mechanical properties and biomimetic apatite mineralization ability of bioceramic composites. These biodegradable and high‐strength composites with outstanding bone regeneration ability present a broad application prospect in repairing ONFH. Wang *et al.* ^[^ ^273^ ^]^ substantiated the excellent drug loading and releasing performance of magnesium‐based layered double hydroxide (LDH) nanosheets. The alendronate (a bone resorption inhibitor used to treat osteoporosis) loading content and encapsulation efficiency of the scaffold reached 197% and 98%, respectively. The abundant loading and continuous release of alendronate promoted the new bone formation and bone mass density effectively. in vivo experiments in repairing ONFH on rabbits showed 1.41 times increase in new bone formation and 1.52 times increase in bone mass density in the necrotic femoral head in the scaffold group compared with the positive control group (treated with autologous bone graft) 8 weeks after the surgery. They also designed a novel bone cement composed of MgAl‐LDH micro‐sheets and poly (methyl methacrylate) (PMMA&LDH). The bone cement showed superior osseointegration performance in vivo and can promote bone regeneration by 18.34‐fold compared to the control group at 2 months postoperatively.^[^ ^277^ ^]^

Apart from magnesium‐contained composites, pure magnesium rods also perform well in repairing ONFH. Katiella *et al.* ^[^ ^274^ ^]^ investigated the effect of magnesium rods loaded with BMP‐2 transfected BMSCs in repairing ONFH on rabbit. Enhanced new bone formation and excellent running ability were observed in the magnesium rod group. The magnesium rod was absolutely absorbed in vivo and caused no damage to important organs. Zheng *et al.* ^[^ ^275^ ^]^ demonstrated that magnesium is harmless and can induce bone and vascularity regeneration. It was found that the combination of magnesium and vitamin C can enhance bone mineral density, bone volume fraction, as well as trabecular number and thickness (Figure 7C).

For clinical application, Zhao *et al.* ^[^ ^276^ ^]^ conducted a pilot randomized controlled trial in 2015 using biodegradable magnesium screws to fix vascularized bone grafting to treat ONFH. After 12 months of follow‐up, they found that patients receiving magnesium screw combined vascularized bone grafting (*n* = 23) showed better imaging and clinical outcomes (higher Harris hip scores) than those who received vascularized bone grafting only (*n* = 25), which was ascribed to the enhanced stability of vascularized bone flaps fixed by magnesium screw and the osteogenic and angiogenic effects of the released Mg^2+^ from the magnesium screw.

In summary, magnesium‐based scaffold is promising in treating ONFH due to Mg^2+^ released from the scaffold was believed to induce bone and blood vessel regeneration.^[^ ^275^ ^]^ However, the current evidence mainly comes from animal experiments and only one pilot study translated the magnesium screw into the clinic to treat ONFH patients.^[^ ^276^ ^]^ Despite encouraging outcomes have been achieved in this clinical trial, before routine clinical applications, further large‐scale randomized controlled trials are needed to validate the efficiency of magnesium‐based scaffold for ONFH treatment.

### 4.2 Tantalum

Tantalum rod can provide subchondral support for necrotic femoral head and has been used to repair ONFH in clinic for many years.^[^ ^278^, ^279^, ^280^, ^281^, ^282^, ^283^, ^284^, ^285^ ^]^ In 2004, a multicenter clinical study employed 98 ONFH patients (113 hips with 17 in stage I and 96 in stage II) to investigate the therapeutic effect of tantalum rods. It turned out that the postoperative average Harris hip score for stage‐II hips was 78 at 24 months and 83 at 48 months. The 2‐year and 4‐year survival rates for stage II patients were 79.1% and 72.5%, and only negligible device‐related complication was reported.^[^ ^286^ ^]^ Veillette *et al.* ^[^ ^287^ ^]^ also used tantalum rods to treat 60 hips in 54 ONFH patients. The 1‐year and 2‐year survival rates reached 81.7% and 68.1%, respectively.

Many studies combine tantalum rods with BMMSCs and vascularized bone grafting to optimize the therapeutic effect.^[^ ^288^, ^289^, ^290^, ^291^, ^292^, ^293^, ^294^ ^]^ Liu *et al.* ^[^ ^295^ ^]^ combined porous tantalum rods with bone graft to treat 149 patients with early‐stage ONFH, and the survival rate reached 95% over 38 months. Zhao *et al.* ^[^ ^296^ ^]^ combined porous tantalum rods with BMMSCs and vascularized iliac grafting to treat 24 end‐stage ONFH patients. With a mean follow‐up time of 64 months, the joint‐preserving success rate was 89.47% for ARCO stage IIIc patients and 75% for ARCO stage IV patients.

However, many tantalum rod‐related failures were also reported.^[^ ^297^, ^298^, ^299^ ^]^ Floerkemeier *et al.* ^[^ ^89^ ^]^ reported that 13 of the 23 necrotic femoral heads receiving tantalum rods treatment need THA within 1.45 years of follow‐up. Tanzer *et al.* ^[^ ^300^ ^]^ carried out a histopathologic analysis of clinically failed tantalum rods and found that the average bone ingrowth distance was only 1.9% (range 0% to 4.4%). Moreover, the failed tantalum rods will complicate the subsequent total hip arthroplasty. The periprosthetic tantalum debris can also increase the incidence of radiolucent line and femoral osteolysis after total hip arthroplasty.^[^ ^301^, ^302^, ^303^ ^]^ Several independent risk factors associated with tantalum rod failure have been identified, including bone marrow edema, corticosteroid intake, age (older than 35 years), and large or laterally located lesions.^[^ ^304^ ^]^

In summary, the effect of porous tantalum rods in treating ONFH is still controversial. Concerning the high failure rate that had been reported in many clinical trials, isolated tantalum rod implantation has been obsoleted in treating ONFH in the clinic in the last few years.^[^ ^305^ ^]^ Meanwhile, the undesirable clinical outcomes also highlight further surface and structural modifications of tantalum rod to increase its efficiency and reduce its failure rate thus bringing it back as a clinical option.

### 4.3 Titanium

With great biocompatibility and low density, titanium has been used as orthopedic implants for many years.^[^ ^306^, ^307^, ^308^ ^]^ However, the mechanical modulus of titanium far exceeds that of cortical bones, which causes an inevitable stress‐shielding effect. With the development of additive manufacturing techniques, porous titanium was fabricated to provide suitable mechanical properties.^[^ ^309^ ^]^ Meanwhile, the porosity can enhance the osteointegration and osteoconduction properties of titanium.^[^ ^310^, ^311^, ^312^, ^313^, ^314^, ^315^, ^316^ ^]^ A 3D porous titanium rod has been designed and achieved encouraging results in repairing ONFH.^[^ ^317^, ^318^, ^319^, ^320^, ^321^, ^322^ ^]^

Wang *et al.* ^[^ ^323^ ^]^ found that the implantation of 3D printed porous titanium rods can increase the maximum load of the femoral head‐necks by 31% compared with core decompression. Micro‐CT and histological evaluation found significantly enhanced bone volume around the porous titanium rod 3 and 6 months postoperatively. Similar results were reported by another study where the researchers fabricated a 3D porous titanium rod with biological lamellar structure to treat early‐stage ONFH sheep (Figure 7D,E).^[^ ^324^ ^]^ Titanium also been combined with vascularized bone graft or osteogenic bioactive molecules to promote its bio‐responsive properties. For example, Zhu *et al.* ^[^ ^317^ ^]^ designed a 3D‐printed titanium scaffold and loaded the scaffold with platelet‐coated gelatin to simulate the extracellular matrix. The bioactive scaffold can induce significant new bone formation and perfectly reconstruct the trabecular bone of the necrotic femoral head. Kang *et al.* ^[^ ^325^ ^]^ combine the recombinant human bone morphogenetic protein (rhBMP)‐2‐loaded titanium implant with autogenous bone graft to repair ONFH in dogs. The radiographic and histological results showed that the implant could completely integrate with the host bone to prevent femoral head collapse. Moreover, Gao *et al.* ^[^ ^318^ ^]^ evaluated the effect of 3D printed porous titanium alloy scaffolds combining daily intraperitoneal injection of trans‐cinnamaldehyde in treating dog ONFH. It turned out the therapy could prevent ONFH progression by promoting osteogenic gene expression, directing osteogenic differentiation, and inhibiting osteoclasts proliferation.

In the clinic, a titanium metal trabecular bone reconstruction system (TMTBRS) was fabricated with titanium powder using the laser sintering technique to treat ONFH.^[^ ^321^, ^326^ ^]^ Chen *et al.* ^[^ ^327^ ^]^ compared the efficiency of the TMTBRS with free vascularized fibular graft in repairing ONFH. The study enrolled 53 patients (63 hips) and the follow‐up time was 7 years. It turned out that TMTBRS has less operation time, reduced bleeding, shorter hospital stays, and better postoperative life quality. However, it is demonstrated that TMTBRS is only recommended to ONFH patients in ARCO IIA and ARCO IIB but not in ARCO IIC.^[^ ^319^ ^]^ The unsatisfied outcomes of titanium in repairing ONFH with larger necrosis volume are mainly ascribed to its poor osteogenesis property. ^[^ ^317^, ^319^, ^325^ ^]^ In conclusion, porous titanium rods with great biocompatibility and superior mechanical properties are promising in treating ONFH, but further large‐scale clinical studies are encouraged to validate their therapeutic efficiency and confirm their long‐term results.

### 4.4 Memory alloys

The most fantastic characteristic of memory alloys is their shape memory effect, which enables them to present different shapes in the implantation process and function period.^[^ ^328^, ^329^, ^330^ ^]^ Wang *et al.* ^[^ ^331^ ^]^ first used a Ni‐Ti memory alloy scaffold to treat ONFH patients in 1998.^[^ ^332^ ^]^ However, the femoral head needs to be opened in the implantation procedure due to the ball shape of the scaffold, which causes significant damage to the femoral head. Recently, Yu *et al.* ^[^ ^333^ ^]^ designed an umbrella‐shaped memory alloy support device with eight supporting umbrella arms and a sleeve (Figure 7F). The scaffold has low elasticity when the temperature is below 37°C, which allows the surgeons to change the scaffold shape into a cylinder before surgery to minimize the implantation damage. As the surrounding temperature increased, the umbrella arms expanded to provide sufficient support for the femoral head. A related clinical trial that enrolled 10 patients (18 hips) with a follow‐up time of 4 to 19 months was conducted and achieved promising results. However, a finite element analysis demonstrated that the surgery will fail if the umbrella arm punctures the formal head, especially when the patient suffers from osteoporosis.^[^ ^334^ ^]^ Therefore, bone mineral density is a significant factor in successfully applying the umbrella‐shaped memory alloy device and should be evaluated preoperatively. Except the above three studies, no other study was identified exploring memory alloys for ONFH treatment. Thus, more studies are needed to verify the efficiency of this innovative material.

## 5 REGENERATIVE THERAPIES FOR ONFH TREATMENT

**FIGURE 8:**
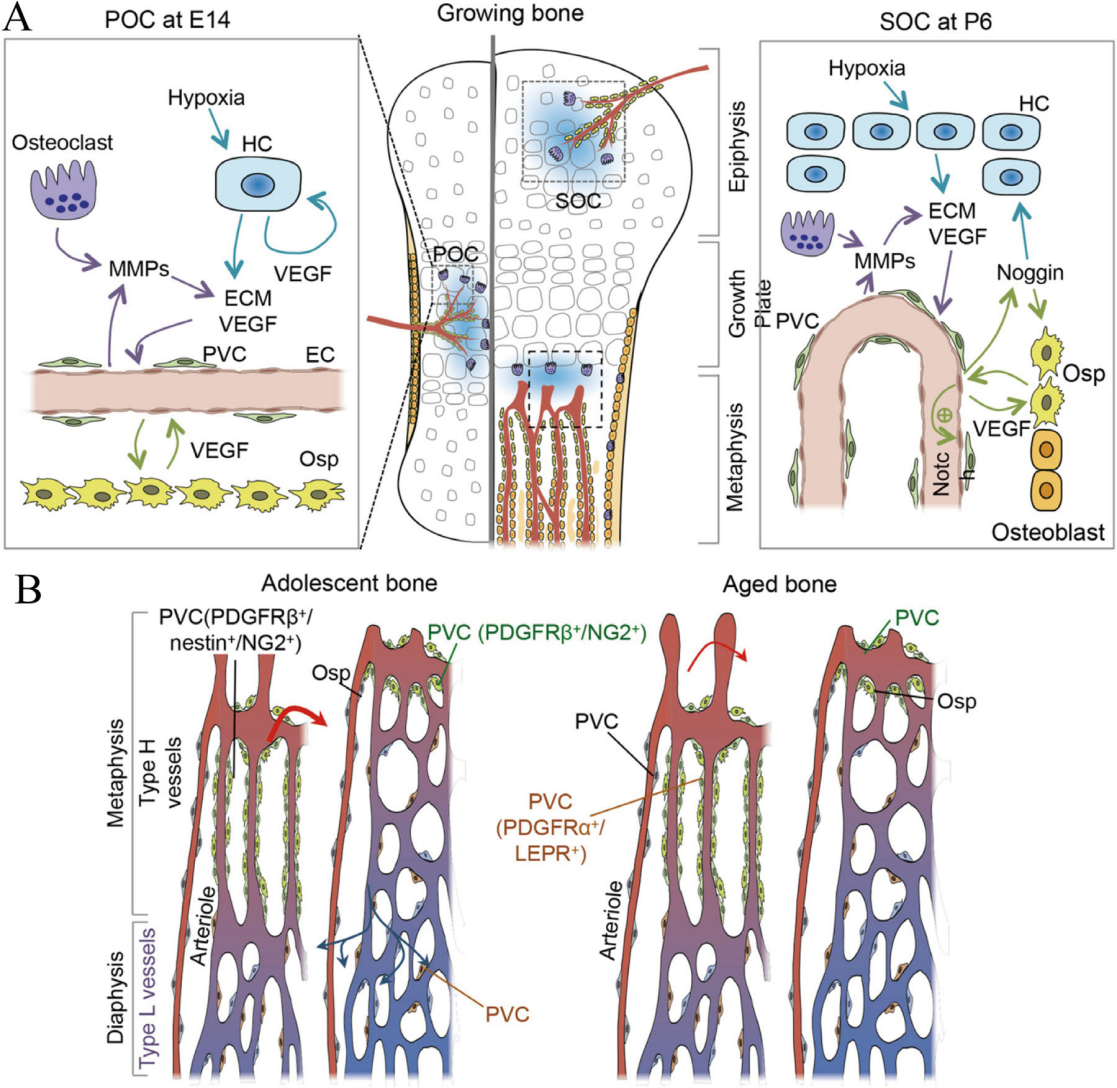
Cells and growth factors involved in orchestrating the genesis and growth of bone and bone vascularity. (A) A variety of cells, growth factors, and signal pathways play an important role in regulating blood vessel and bone coupling generation during the process of endochondral ossification. (B) Perivascular cells differ in adolescent bone and aged bone, which is associated to reduced blood flow and bone generation in aged bone. Reproduced with permission.^[^ ^335^ ^]^ Copyright 2017, The American Association for the Advancement of Science.

**FIGURE 9:**
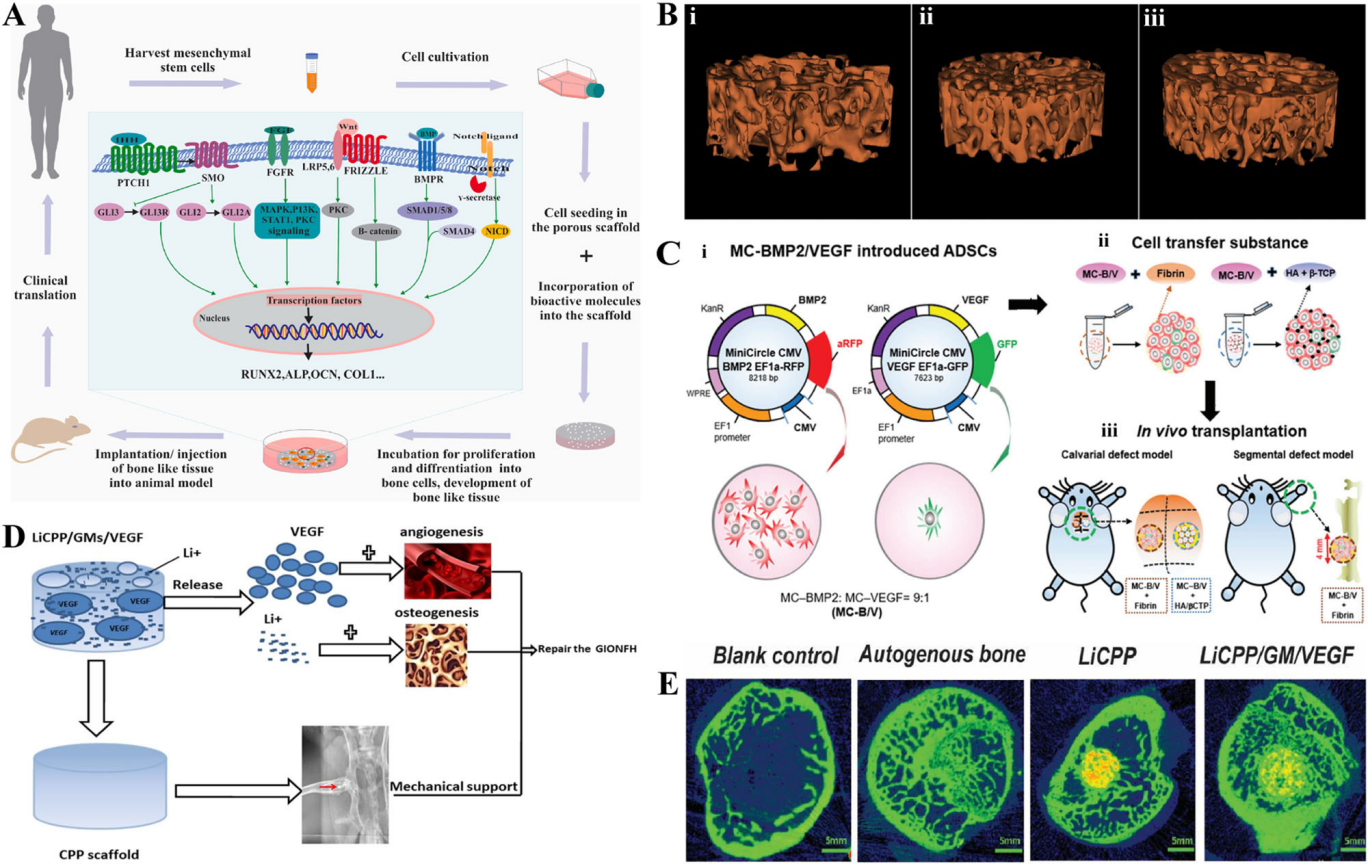
Biological regenerative therapies for ONFH. (A) Schematic illustration of the biological regenerative therapies in bone tissue engineering. Reproduced with permission.^[^ ^337^ ^]^ Copyright 2021, Elsevier. (B) Magnified interest region of the femoral head of the ONFH model group (i), ONFH BMMSC transplantation group with 20% O_2_ (ii), and ONFH BMMSC transplantation group with 2% O_2_ (iii). The best three‐dimensional trabecular bone structure was observed in the ONFH BMMSC transplantation group with 2% O_2_. Reproduced with permission.^[^ ^351^ ^]^ Copyright 2015, Elsevier. (C) Schematic illustration of gene therapy fabricating and transplanting BMP‐2/VEGF (B/V)‐introduced stem cells to induce blood vessel and bone regeneration in vivo. Reproduced with permission.^[^ ^366^ ^]^ Copyright 2019, Royal Society of Chemistry. (D) VEGF‐loaded CPP scaffold with potent angiogenesis and osteogenesis properties was designed and fabricated. Reproduced with permission.^[^ ^200^ ^]^ Copyright 2019, IOP Publishing, Ltd. (E) Significantly enhanced new bone formation in the femoral head defects was observed in the VEGF‐loaded CPP scaffold group compared with control group. Reproduced with permission.^[^ ^200^ ^]^ Copyright 2019, IOP Publishing, Ltd. MC, minicircle vectors; ASCs, adipose stem cells; RFP, red fluorescent protein; GFP, green fluorescent protein.

Bone and vascularity regenerations are crucial in repairing the necrotic femoral head. Native cells and growth factors play an important role in orchestrating the genesis and growth of bone and bone vascularity (Figure 8).^[^ ^335^ ^]^ Thus, biological regenerative therapies that transplant cells and bioactive molecules to induce new bone and vascularity formation show a bright prospect in treating ONFH.^[^ ^30^, ^336^ ^]^ Biomaterial scaffolds are often involved in biological regenerative therapies to provide 3D support structures and adhesion sites (Figure 9A).^[^ ^337^, ^338^, ^339^, ^340^, ^341^ ^]^ Here, we summarize and evaluate the biological regenerative therapies used in ONFH treatment.

### 5.1 Stem cells

The decreased osteogenic progenitor cells partly account for the insufficient substitution of bone remodeling after osteonecrosis,^[^ ^342^ ^]^ so stem cell‐based therapies have attracted much attention in repairing ONFH for more than 50 years.^[^ ^343^, ^344^ ^]^ Various cell sources have been used in repairing ONFH, among which BMMSCs are the most widely exploited cells for their easy access and great osteogenic ability.^[^ ^345^, ^346^, ^347^ ^]^ Gangji *et al.* ^[^ ^348^ ^]^ demonstrated that the implantation of autologous BMMSCs can effectively repair early‐stage ONFH. Reduced necrotic volume and improved survivorship were observed in the stem cells‐transplanted patients. Li *et al.* ^[^ ^349^ ^]^ intravenously injected BMMSCs to treat rabbit ONFH. They found that the therapy can upregulate osteogenic and angiogenic mRNA transcription and protein expression, thus promoting vascularity and bone regeneration. Moreover, many studies precondition stem cells to further improve their therapeutic effects.^[^ ^350^, ^351^, ^352^ ^]^ Fan *et al.* ^[^ ^351^ ^]^ cultured BMMSCs in a hypoxia environment before implantation. A better 3D structure and enhanced trabecular bone integrity were found in the hypoxia pretreated BMMSCs group (Figure 9B). Zhao *et al.* ^[^ ^352^ ^]^ conducted a randomized controlled trial involving 100 patients (104 hips) with a follow‐up time of 5 years using BMMSCs to treat ONFH. They first cultured and expanded autologous BMMSCs In vitro to achieve a greater cell number. The proliferated BMMSCs were implanted to the necrotic femoral head and were found to have significantly alleviated clinical symptoms and decreased the necrotic volume of the femoral head compared with pristine stem cells.

Biomaterial scaffolds can provide 3D structures and resemble natural extracellular matrix to facilitate stem cell adhesion and proliferation.^[^ ^129^, ^130^, ^139^, ^141^, ^242^, ^288^, ^353^, ^354^, ^355^, ^356^, ^357^, ^358^ ^]^ Yamasaki *et al.* ^[^ ^130^ ^]^ loaded BMMSCs onto a porous HA scaffold to treat ONFH. Compared with the control group that only implanted a porous HA scaffold, the BMMSCs group showed reduced necrotic volume and delayed femoral head collapse. Hernigou *et al.* ^[^ ^359^ ^]^ followed 189 hips for 5 to 10 years and found the autologous bone marrow contained graft has better clinical and radiographic outcomes than the simple core decompression. However, not all patients with early‐stage ONFH are suitable for stem cell therapy. Disease type (according to the China‐Japan Friendship Hospital classification system) is an important factor for the therapeutic effect of BMMSCs, type L2 showed a significantly higher failure rate (60.0%) than type L1 and L3.^[^ ^360^ ^]^ Moreover, the multipotent and heterogeneous nature of stem cells makes it difficult to monitor and regulate the therapy.^[^ ^361^ ^]^ Limited self‐renewal property and unstable activity of stem cells also restrict their application.^[^ ^362^ ^]^ In addition, the cell number required for effective bone regeneration varies with the different cell sources and scaffold properties.^[^ ^363^ ^]^ Therefore, more efforts are needed to confirm the indications and cell number‐dependent efficiency of cell‐based therapies.

### 5.2 Bioactive molecules

The process of physiological bone repairing is extremely complicated, in which bioactive molecules play a vital role in regulating signal pathways and cell behavior.^[^ ^363^, ^364^, ^365^ ^]^ The combination of bioactive factors with 3D scaffolds can effectively simulate in vivo microenvironments that facilitate bone and vascularity regeneration.^[^ ^366^ ^]^ Currently, three methods were used to apply bioactive molecules in bone tissue engineering, namely cell therapy, gene therapy, and cytokine therapy.^[^ ^367^ ^]^ Cell therapy co‐cultures bioactive molecules and stem cells to induce the osteogenic differentiation of stem cells, and then transplants the stem cells to defected bone. Gene therapy transduces bioactive molecule genes to cells in vivo or in vitro to promote osteogenic protein expression after implantation (Figure 9C),^[^ ^366^ ^]^ whereas cytokine therapy directly delivers bioactive molecules in bone defect areas through a well‐designed delivery system. Cytokine therapy is considered the most promising therapy for its convenience and safety. Many bioactive molecules have been proved effective in bone tissue engineering,^[^ ^368^, ^369^, ^370^, ^371^, ^372^, ^373^, ^374^, ^375^, ^376^, ^377^, ^378^, ^379^, ^380^, ^381^ ^]^ among which BMP‐2 and VEGF are the most widely used in repairing ONFH for their potent osteogenesis and angiogenesis properties.^[^ ^253^, ^325^, ^382^, ^383^ ^]^

BMP‐2 is a dimeric protein that can induce stem cells to differentiate osteocyte and chondrocyte lineages.^[^ ^384^ ^]^ The osteocytes in the necrotic femoral head usually express significantly lower BMP‐2 mRNA and protein,^[^ ^385^ ^]^ thus exogenous BMP‐2 can help restore the natural trabecula structure and mechanical strength of the femoral head.^[^ ^386^ ^]^ Zhu *et al.* ^[^ ^225^ ^]^ found natural polymer composites loaded with BMP‐2 microspheres could promote calcium deposition and new bone formation of the necrotic femoral head in steroid‐induced ONFH rats. Katiella *et al.* ^[^ ^387^ ^]^ entrapped BMP‐2 into the PLGA‐HA microsphere and found that the released BMP‐2 from the microsphere significantly induced bone regeneration and reduced osteonecrosis volume. Gene therapy that transplants BMP‐2 transfected BMMSCs to the femoral head was also explored.^[^ ^274^ ^]^ Tang *et al.* ^[^ ^388^ ^]^ used β‐TCP loaded (BMP‐2)‐gene‐modified BMMSCs to repair ONFH in goats. A significantly enhanced new bone formation and better mechanical properties were obtained in the (BMP‐2)‐gene‐modified BMMSCs group. Peng *et al.* ^[^ ^386^ ^]^ loaded BMP‐2‐expressed BMMSCs in DBM and observed increased bone volume and higher neovascularization density around the scaffold in a dog ONFH. However, heterotopic ossification has also been reported and should be noticed when applying high dose BMP‐2.^[^ ^389^ ^]^

In addition to osteogenesis, angiogenesis is another essential parameter in repairing ONFH. As the most important angiogenesis factor, VEFG can promote endothelial cell proliferation and migration, thus enhancing new vascularity formation.^[^ ^390^, ^391^, ^392^, ^393^ ^]^ VEFG also plays an essential role in bone tissue formation and growth, especially in the process of endochondral ossification.^[^ ^394^, ^395^, ^396^ ^]^ Increased VEGF protein and mRNA expression are found in the epiphyseal cartilage of the necrotic femoral head, which can facilitate neovascular formation.^[^ ^397^, ^398^ ^]^ Luo *et al.* ^[^ ^200^ ^]^ loaded VEGF‐wrapped gelatin microsphere in a CPP scaffold to realize sustainable VEGF release, which was proved to increase angiogenic and osteogenic factors expression and help repair ONFH (Figure 9D,E). Lee *et al.* ^[^ ^337^ ^]^ designed VEGF‐ and BMP‐2‐transfected adipose stem cells and achieved rapid osteogenesis and angiogenesis in an avascular environment. Cao *et al.* ^[^ ^399^ ^]^ implanted a VEGF‐expressed recombinant plasmid‐loaded scaffold to the necrotic femoral head. Higher VEGF and collagen I expression along with enhanced new bone formation and capillary regeneration were observed in the necrotic femoral head.

In summary, bioactive molecules have great pharmacological potential, and the corresponding delivery system dictates the effective and safe release of these molecules. However, the high cost and complex regulatory requirements of bioactive molecules still hinder their application and need to be solved.^[^ ^400^ ^]^

### 5.3 Latest developments to improve regenerative therapies

Several novel regenerative therapies emerged in bone tissue engineering in the last few years. First, stem cell‐derived products such as secretome and extracellular vesicles that contain genetic material and various bioactive factors playing an important role in intercellular interaction attract increasing attention in bone tissue engineering. With potent osteogenic capabilities, secretome and extracellular vesicles hold better accessibility and biosecurity as well as fewer ethical issues compared with original stem cells.^[^ ^401^ ^]^ Regarding ONFH, it was reported that BMSCs‐derived extracellular vesicles can promote the osteogenic and angiogenic differentiation but inhibit the adipogenic differentiation of host BMSCs, thus preventing the progression of ONFH.^[^ ^402^ ^]^ Moreover, genetically engineered EVs can over‐express target bioactive molecules to further enhance their therapeutic effects for ONFH.^[^ ^402^ ^]^ Second, since an essential challenge in bone tissue engineering is to recapitulate the structure and composition of natural bone using materials, bone organoids became a research focus in recent years. By bioprinting the bioactive molecules, cellular aggregates, and microtissues, bone organoids can perfectly mimic natural bone at various scales and hold the promise to revolutionize the treatment of bone diseases including ONFH.^[^ ^403^, ^404^ ^]^ For example, Gabriella *et al.* ^[^ ^404^ ^]^ used periosteum‐derived cells to produce microspheroids that are capable to differentiate into callus organoids. The callus organoids can further spontaneously bioassemble into bone organoids that can heal critical‐sized long bone defects. Third, vehicles that can deliver drugs and bioactive factors in a spatially/temporally controlled manner have emerged as a promising therapy in tissue engineering. The environment‐responsive vehicles can sense the changes in the microenvironment such as ischemia and inflammation and accordingly release drugs or factors to adapt to the disease states or changes.^[^ ^405^ ^]^ For instance, Messina *et al.* ^[^ ^405^ ^]^ designed a microfluidic chip refined with plasmonic nanotubes that can spatially, temporally, and quantitatively deliver arbitrary molecules into selected single cell and cell group, proposing a promising technique for cell‐level diagnosis and therapy. In conclusion, these state‐of‐the‐art advances in bone tissue engineering provide more possibilities for ONFH treatment and are worthy of further study.

## 6 CONCLUSION AND PERSPECTIVE

As a progressive disease, ONFH has a high morbidity and disability rate and causes a tremendous socioeconomic burden. Although total hip arthroplasty can effectively ameliorate the pain and reconstruct the hip joint function in ONFH patients, prosthesis revision or a second surgery may be required when prosthesis wear occurs. Efforts have been made to circumvent total hip arthroplasty by preventing the femoral head from collapsing. For example, core decompression has become the most popular femoral head‐preserving surgery that can eliminate femoral head pressure, ameliorate tissue hypoxia, and reduce pain. However, core decompression cannot reconstruct the mechanical support for the necrotic femoral head or induce new bone and vascularity formation. Autologous bone grafting has been employed to fill the tunnel drilled in core decompression surgery to provide subchondral support and induce bone and vascularity regeneration, but the involvement of autologous bone grafting also brings many complications. To break out of this dilemma, bone tissue engineering has been extensively developed to make up for the deficiencies of these surgeries and improve the prognosis of ONFH patients. Over the past few decades, bone tissue engineering has made great strides in achieving high‐efficiency ONFH treatment.

Despite many advancements having been achieved in ONFH‐repairing scaffolds, numerous concerns remain before their successful clinical translation. Here, we concluded the primary gaps between material‐design and clinical translation and then proposed potential approaches to surmount these gaps. First, the mechanical properties of most scaffolds explored in animal experiments do not perfectly match the natural human bone, resulting in insufficient subchondral support or stress shielding effect. It is recommended to adjust the mechanical properties of scaffolds by optimizing the internal structure of scaffolds with advanced manufacturing techniques or combining biomaterials with different mechanical properties. In addition, attention should be paid to the patients’ underlying diseases such as osteoporosis, which also affects the design of mechanical properties of materials. Second, unsatisfactory osteogenesis and osteointegration properties of designed scaffolds make them prone to loosen and displace upon the patient moves. Surface modification and the porous structure can facilitate bone ingrowth and reinforce the integration between the scaffolds and natural bone. Loading stem cells and bioactive molecules is another promising solution. In addition, enhanced angiogenic properties are also essential to induce new vessel formation that facilitates bone regeneration. Third, degradability of the scaffolds is a desired property to avoid additional surgery, but the degradation rate should match bone remodeling to provide continuous subchondral support. At the same time, the degradation byproducts should not alter the physiochemical characteristics of the surrounding microenvironment such as pH and osmotic pressure. Thus, biocompatible biomaterial with adjustable degradation rates is preferred. Finally, for clinical use, the sterilization method, expenses, and difficulty of commercialization of the scaffold should also be considered before design.

Apart from material‐design consideration, scalable fabrication technologies such as additive manufacturing, electric‐field‐assisted technique and aspiration‐assisted bioprinting are indispensable for the fabrication of elaborate scaffolds.^[^ ^28^, ^406^ ^]^ Additive manufacturing, also called 3D printing, is a promising fabrication technique with the capability to customize the shape, pore size, and mechanical property of the scaffold, offering extraordinary opportunities for precise preparation of biomaterials toward ONFH treatment.^[^ ^309^, ^407^ ^]^ 3D bioprinting as a part of additive manufacturing can print disease models of ONFH for preclinical drug testing. With unprecedented capability of manipulating fine structure of printed scaffold, 3D bioprinting was widely explored to recapitulate more anatomically‐similar bone grafts, which are also expected to be applied in ONFH treatment.^[^ ^28^ ^]^ Moreover, rapidly developing nanotechnology creating more ingenious biomaterials also bring us more opportunities to construct functional biomimetic bone. Vehicles that can spatially/temporally control drugs and factors delivery have also been widely exploited to promote precise medicine and intelligent medicine.^[^ ^405^, ^408^ ^]^ Moreover, the establishment of a bipedal animal ONFH model is encouraged to better simulate and understand the natural progressive process of ONFH in human beings.^[^ ^252^ ^]^ At last, a deeper understanding of the underlying physiopathologic mechanism of ONFH, especially the complex interaction between cells, bioactive molecules, and the specific signaling pathways will guide us to optimize the current therapies. Stem cell engineering that can realize over‐ or under‐express bioactive molecules and up‐ or down‐regulate signal pathways that affect the pathogenesis of the ONFH may play an important role in this process.^[^ ^409^ ^]^ It is believed that the advancements of the material‐design concept as well as further identification of the ONFH pathogenic mechanism will bring us unprecedented opportunities to design ever more sophisticated scaffolds for repairing ONFH.

## CONFLICT OF INTEREST

The authors declare no conflict of interest.
